# Contraction of the rigor actomyosin complex drives bulk hemoglobin expulsion from hemolyzing erythrocytes

**DOI:** 10.1007/s10237-022-01654-6

**Published:** 2022-11-10

**Authors:** Ryo Shirakashi, Dmitri Sisario, Danush Taban, Tessa Korsa, Sophia B. Wanner, Julia Neubauer, Cholpon S. Djuzenova, Heiko Zimmermann, Vladimir L. Sukhorukov

**Affiliations:** 1 Institute of Industrial Science, The University of Tokyo, Tokyo, Japan; 2 Department of Biotechnology and Biophysics, Biocenter, University of Würzburg, Würzburg, Germany; 3 Fraunhofer Institute for Biomedical Engineering (IBMT), Sulzbach, Germany; 4 Department of Radiation Oncology, University Hospital of Würzburg, Würzburg, Germany; 5 Department of Molecular and Cellular Biotechnology, Saarland University, Saarbrücken, Germany; 6 Faculty of Marine Science, Universidad Católica del Norte, Coquimbo, Chile

**Keywords:** Electroporation, Cell velocimetry, Hemoglobin jet, Non-muscle myosin, Echinocytes, Cytoskeleton

## Abstract

Erythrocyte ghost formation via hemolysis is a key event in the physiological clearance of senescent red blood cells (RBCs) in the spleen. The turnover rate of millions of RBCs per second necessitates a rapid efflux of hemoglobin (Hb) from RBCs by a not yet identified mechanism. Using high-speed video-microscopy of isolated RBCs, we show that electroporation-induced efflux of cytosolic ATP and other small solutes leads to transient cell shrinkage and echinocytosis, followed by osmotic swelling to the critical hemolytic volume. The onset of hemolysis coincided with a sudden self-propelled cell motion, accompanied by cell contraction and Hb-jet ejection. Our biomechanical model, which relates the Hb-jet-driven cell motion to the cytosolic pressure generation via elastic contraction of the RBC membrane, showed that the contributions of the bilayer and the bilayer-anchored spectrin cytoskeleton to the hemolytic cell motion are negligible. Consistent with the biomechanical analysis, our biochemical experiments, involving extracellular ATP and the myosin inhibitor blebbistatin, identify the low abundant non-muscle myosin 2A (NM2A) as the key contributor to the Hb-jet emission and fast hemolytic cell motion. Thus, our data reveal a rapid myosin-based mechanism of hemolysis, as opposed to a much slower diffusive Hb efflux.

**Supplementary Information:**

The online version contains supplementary material available at 10.1007/s10237-022-01654-6.

## Introduction

Erythrocytes or red blood cells (RBCs) are the most abundant cell type in the human body. RBCs are discoid, biconcave cells, whose main function is oxygen transport via their hemoglobin-rich cytoplasm. Their shape and unique mechanical properties, including deformability, elasticity, and durability, enable RBCs to squeeze through capillaries and microfluidic channels with a diameter much smaller than their own (Mohandas and Gallagher, 2008; Wu et al., 2015; Asaro and Zhu, 2020). The stability and elastic deformability of RBCs rely on the composite membrane, consisting of the lipid bilayer laminated by the spectrin cytoskeleton (Mauer et al., 2018; Turlier et al., 2016), along with a large excess of membrane area over that of a sphere of the same volume (Brooks and Evans, 1987). Glucose and ATP depletion in vitro and in vivo, *e.g.,* upon storage and aging, have strong impact not only on the RBC morphology, such as discocyte-to-echinocyte transition but also on the elastic cell properties (Braunmüller et al., 2012; Feo and Mohandas, 1977; Putter and Seghatchian, 2017; Xu et al., 2018).

In sharp contrast to the remarkable mechanical stability of discoid RBCs in physiological media, RBCs are highly fragile to hypotonicity and other stresses, such as electroporation, photodynamic damage, etc., which cause osmotically driven cell swelling and hemolysis (Kinosita and Tsong, 1977; Marsden et al., 1981). Unlike normal discoid RBCs, ATP-depleted echinocytes are strikingly rigid cells with an increased resistance to osmotic swelling and lysis (Dreher et al., 1978; Clark et al. 1981). Hemolysis, *i.e.,* the release of hemoglobin (Hb), involves the formation of hemolytic hole(s) or pore(s) in the bilayer. During hemolysis, RBCs convert to Hb-depleted membrane shells, commonly known as “ghosts”. The inherent osmotic fragility of RBCs is due to the limited amount of bilayer material (Lim et al., 2008).

Hemoglobin release can occur either over the entire cell surface via multiple membrane pores or through a single pore (Hoffman, 1992). A number of studies even show a non-diffusive mechanism of hemolysis, such as ejection of the pressurized cytosol. Thus, independent of the hemolyzing agent, the onset of hemolysis occurs abruptly and the initial efflux rate of Hb is much higher than expected for its free diffusion (Heedman, 1958). Similarly, photohemolysis begins suddenly with Hb emission in a rapidly escaping jet-like cloud (Marsden, 1995). Some Hb-jets were capable of propelling emitting cells in the opposite direction (Marsden et al., 1981). Likewise, hypotonic stress causes ejection of the cytoplasm from hemolyzing RBCs leading to fast cell movement (Zade-Oppen, 1998). Electrofused RBCs also emit transient cytosol jets (Baumann, 1999), indicative of high cytosolic pressure. All these studies point to Hb-jets as a common phenomenon during swelling-mediated hemolysis. Consistent with the Hb-jet driven cell motion (Zade-Oppen, 1998), hypotonically produced RBC ghosts possess a single hemolytic hole with a radius of 10–20 nm (Lieber and Steck, 1989), permeable for Hb molecules with a Stokes’ radius of 3.1 nm (Doster and Longeville, 2007).

The mechanisms of hemolysis and related mechanical properties of erythrocytes have been the subject of numerous experimental and theoretical studies (Zhang and Brown, 2008; Klöppel and Wall, 2011; Boal, 2012; Faghih and Sharp, 2019; Wu et al., 2019; Craven et al., 2019; Asaro and Zhu, 2020; Horobin et al., 2020; Karandeniya et al., 2022). A recent study identified hemolysis as a key event in the clearance and turnover of billions of senescent erythrocytes per day in the spleen (Klei et al., 2020). Yet, despite the physiological significance of hemolysis, the underlying molecular mechanisms of Hb-jet emission have neither been studied nor analyzed quantitatively.

Here, we explore the rapid hemolysis of human RBCs initiated by electroporation. Electroporation is well known to cause hemolysis by inducing several hundreds of sub-nm-sized electropores per cell (Sowers and Lieber, 1986; Tekle et al., 1994). As a result, the cell membrane becomes permeable to otherwise impermeant inorganic ions and small organic solutes, such as glucose, sorbitol, sucrose, ATP, etc. (Kinosita et al., 1988; Zimmermann et al., 2000). Driven by concentration gradients, the efflux of intracellular solutes and influx of external solutes through electropores lead, respectively, to osmotic cell shrinkage or swelling (Shirakashi et al., 2002; Sözer et al., 2017). While electroporation depletes small cytosolic solutes, such as K^+^, Cl^−^, ATP, etc. (Saulis and Saulė, 2012), the enduring influx of an abundant extracellular solute, such as sorbitol used in our experiments, along with the excess osmotic pressure due to presence of large membrane impermeable colloidal solutes, including hemoglobin and other proteins (Gary-Bobo and Solomon, 1968), causes RBC swelling to the critical volume and hemolysis (Kinosita and Tsong, 1977).

Among the known hemolytic agents, electroporation appears to be the most suitable approach to analyze the hemolytic cell motion. Unlike hypotonic and photodynamic stresses, which generate membrane pore(s) randomly over the entire cell surface, the electropore formation preferentially occurs at the cell poles facing the electrodes (Kinosita et al., 1988; Tekle et al., 1994) and is naturally aligned with the focal plane of a microscope thus providing optimal conditions for cell tracking during hemolyis. Finally, electrohemolysis is induced under *isotonic* conditions, which, unlike hypotonic stress, does not require any medium exchange, thus enabling unperturbed cell tracking velocimetry.

Using high-speed video microscopy, we analyzed the morphological changes of cells triggered by electroporation and the hemolytic cell motion. Our (osmo-) elastic membrane models allowed us to relate the Hb-jet-driven cell motion to the cytosolic pressure generation and elastic contraction of the RBC membrane. We showed that the contributions of the bilayer and the bilayer-anchored spectrin cytoskeleton to the hemolytic cell motion are negligible. Consistent with these findings, the results of our biochemical experiments, involving extracellular ATP and the myosin inhibitor blebbistatin, demonstrate that the low abundant non-muscle myosin 2A (NM2A) (Smith et al., 2018) is a key contributor to the Hb-jet emission and fast hemolytic cell motion.

## Materials and Methods

### Reagents and buffers

Phosphate-buffered saline (PBS) was prepared by dilution of the 10-times concentrated Dulbecco’s PBS (without CaCl_2_ and MgCl_2_, Sigma, #D1408) with Millipore water. RBC buffer consisted of PBS and 1 mg/ml bovine serum albumin (BSA, Sigma, #A3983). Sorbitol was purchased from Merck (product #6213.1). ATP and blebbistatin were purchased from Sigma, product number A6419 and B0560, respectively.

### RBC sample preparation

RBCs were obtained from three healthy consenting donors by finger pricking. RBC stock suspension was prepared by 20-fold dilution of ~ 50 µl blood in RBC-buffer. This stock suspension of ~ 2.5 × 10^8^ cells/ml was kept on ice for no longer than 2 h prior to experiments. Shortly before electroporation, 50 µl of the stock cell suspension was diluted in 5 ml of an isotonic sorbitol solution to a cell density of ~ 2 × 10^6^ cells/ml. Cell suspension in sorbitol had a conductivity of ~ 100 µS/cm, which was measured using a conductometer LAQUAtwin B-771 (Horiba, Kyoto, Japan). Blebbistatin was dissolved in dimethyl sulfoxide (DMSO, Sigma-Aldrich) to make a 7 mM stock solution. This stock solution was added to an RBC suspension to yield a final concentration of 70 µM blebbistatin and 1% DMSO. The same concentration of DMSO (without blebbistatin) was used in control experiments. After the addition of blebbistatin and/or DMSO, the RBCs were incubated for 1 h before electroporation.

### Microscopy and video imaging systems

Cell movement was captured with the digital CCD cameras uEye 2240 and uEye-UI 3060CP-C-HQR2 (IDS, Obersulm, Germany). The cameras were attached to a phase-contrast microscope (Axiophot, Zeiss, Oberkochen, Germany) or alternatively to a fluorescence microscope (BX51 Olympus, Hamburg, Germany). Video recordings were acquired at 15 to 100 frames per second (fps), starting ~ 2 s before and up to 10–20 s after electroporation.

### Electroporation chamber and pulse generator

The electroporation chamber consisted of two cylindrical stainless-steel electrodes with a diameter of 200 µm (Supplemental Fig. S1). The electrodes were mounted in parallel at a distance of 200 µm on a microscope slide. An aliquot of RBC suspension (~ 50 µl) was pipetted between the electrodes and was covered by a glass coverslip. Hemolysis was induced at room temperature (~ 22 °C) by a single rectangular pulse of 3.5 kV/cm strength and 40 µs duration by using the Eppendorf-Multiporator (Eppendorf, Hamburg, Germany).

### Velocimetry of hemolyzing RBCs

Tracking of hemolyzing RBCs was performed using the image-processing software ImageJ (NIH, Bethesda, MD). Briefly, in each video sequence, several regions of interest (ROIs) containing single RBCs were randomly selected and extracted as separate image stacks. Typically, image stacks contained up to 1000–2000 frames separated by 10 ms, including ~ 2 s before electroporation. Automated cell tracking and projected cell area (*A*_proj_) measurement were implemented with an in-house developed ImageJ macro (for detail see Supplement, Fig. S2).

Trajectories of individual cells were plotted by setting the position of each cell at the onset of hemolysis to the origin of a Cartesian coordinate system. From the acquired trajectories, cell displacements between two successive frames were calculated as ${\Delta D}_{i}= Sqrt({\left({x}_{i}-{x}_{i-1}\right)}^{2}+{\left({y}_{i}-{y}_{i-1}\right)}^{2})$, where *x*_*i*_ and *y*_*i*_ are the *x*- and *y*-positions of the cell in frame *i*. The cell velocity *v*_cell_(*t*) in µm/s was calculated as *v*_cell_(*t*) = Δ*D*_i_(*t*)/Δ*t*, where the time interval between two frames Δ*t*. Usually, tracking of 40–50 hemolyzing RBCs was performed in at least five independent experiments. Only cells exhibiting linear trajectories were evaluated to generate the velocity and displacement curves. To allow for the cell-to-cell variability in the time lag between electroporation and the onset of hemolysis, the time courses of cell velocity and displacement were averaged by synchronizing the time points of maximum cell acceleration.

### Theory: the minimal elastic model of hemolytic cell contraction and motion

To gain insight into the biomechanics of the self-propelled RBC motion during hemolysis, we devised a minimal elastic model, in which cell motion is driven by ejection of pressurized cytosol. The cytosolic pressure can be generated by tension/contraction of either the membrane bilayer or the cytoskeleton, both of which are integrated into our model via their respective elastic constitutive equations. Using a more general osmo-elastic model (*see* Supplement), we show that the osmotic water influx through the membrane has little effect on the hemolytic cell motion. Therefore, the osmotic flux during hemolysis is neglected in the minimal model. The model assumes spherical cell geometry and comprises Eqs. 1–6 given below.

The differential Eq. 1 is the ruling equation of motion of a jet-propelled, variable-mass body travelling through a viscous fluid medium. Derived in detail in the Supplement, Eq. 1 accounts for the force balance between the Hb-jet thrust and the viscous drag acting on the cell during hemolytic motion. A similar equation applies to the motion of aquatic animals, such as squids, that use jet propulsion as a means of locomotion (Johnson et al., 1971).

1 $$ \underbrace {{m_{{{\text{cell}}}} \frac{d}{dt}\left( {v_{{{\text{cell}}}} } \right)}}_{{\begin{array}{*{20}c} {{\text{inertial}}\;{\text{term}}} \\ {{\text{of}}\;{\text{the}}\;{\text{cell}}} \\ \end{array} }} = \underbrace {{\rho v_{{{\text{jet}}}}^{2} S_{{{\text{hole}}}} }}_{{\begin{array}{*{20}c} {{\text{thrust}}\;{\text{force}}} \\ {{\text{by}}\;{\text{the}}\;{\text{Hb}}\;{\text{jet}}} \\ \end{array} }} - \underbrace {{6\pi a\eta v_{{{\text{cell}}}} }}_{{\begin{array}{*{20}c} {{\text{viscous}}\;{\text{drag}}} \\ {{\text{force}}} \\ \end{array} }} $$

where the cell mass ${m}_{cell}=4\pi {a}^{3}\rho /3$ for a spherical cell of radius *a* and density *ρ* ≈ 1 kg/m^3^. Symbols *v*_cell_ and *v*_jet_ stand, respectively, for the cell and Hb-jet exit velocities. Symbol *S*_hole_ denotes the cross-section area of the hemolytic hole of radius *r*_hole_ (${S}_{\mathrm{hole}}=\pi {r}_{\mathrm{hole}}^{2}$). Symbol $\eta$ stands for the viscosity of the surrounding solution. For calculations, we used the viscosity value of *η* = 1 mPa⋅s reported in the literature for isotonic 300 mM solutions of sorbitol and other monomeric sugar alcohols (Tu et al., 2022; Sukhorukov et al. 1993).

Equation 2 implies that the RBC volume decreases during hemolysis only via the convective Hb-jet outflow through the hemolytic hole:

2 $$\underbrace {{\frac{d}{dt}\left( {\frac{4}{3}\pi a^{3} } \right)}}_ {{\begin{array}{*{20}c} {{\text{cell}}\;{\text{volume}} } \\ {{\text{change}}} \\ \end{array} }} = 4\pi a^{2} \frac{da}{{dt}} = \underbrace {{-v_{jet} S_{h} }}_ {{\begin{array}{*{20}c} {{\text{volume}}\;{\text{flux}} } \\ {{\text{in}}\;{\text{cytosol}}\;{\text{jet}}} \\ \end{array} }} $$

Equation 3 describes an exponential expansion of the hemolytic hole at the onset of hemolysis. Pore expansion is caused by the mechanical tension in the bilayer (Brochard-Wyart et al., 2000; Lim, 2008) merging upon cell swelling to the critical hemolytic volume:

3 $$ S_{{{\text{hole}}}} = {\uppi }r_{{{\text{hole}}}}^{2} = S_{{{\text{hole}}}}^{{{\text{min}}}} + \overbrace {{\left( {S_{{{\text{hole}}}}^{{{\text{max}}}} - S_{{{\text{hole}}}}^{{{\text{min}}}} } \right)\left( {1 - \exp ( - t/\tau_{{{\text{hole}}}} } \right)}}^{{\text{hemolytic hole expansion}}} $$

where ${S}_{\mathrm{hole}}^{\mathrm{min}}$ and ${S}_{\mathrm{hole}}^{\mathrm{max}}$ are, respectively, the areas of the prehemolytic and hemolytic holes. Symbol *τ*_hole_ denotes the time constant of hole expansion at the onset of hemolysis.

The Bernoulli equation (Eq. 4) relates the Hb-jet exit velocity to the cytosolic pressure (∆*p*_cyt_), assuming inviscid cytosol jet flow through the hemolytic hole:

4 $${v}_{jet}=\sqrt{2\Delta {p}_{\text{cyt}}/\rho }$$

We also tested the Roscoe equation (Roscoe, 1949) for a viscous jet flow (Supplementary Fig. S5).

And finally, the bilayer and the cytoskeleton, are integrated into our model via two different elastic constitutive equations (Eqs. 5 and 6). Based on the area elasticity theory (Helfrich, 1973), Eq. 5 regards the RBC as a sphere enclosed by a 2D elastic *shell* (i.e., membrane bilayer) and relates Δ*p*_cyt_ to the areal elasticity modulus of the bilayer *K*_shell_. The cell radii *a* and *a*_0_, correspond, respectively, to an expanded tense (e.g., spherocyte) and a fully relaxed/contracted membrane with *zero tension* and a negligible cytosolic pressure (e.g. ghost).

5 $$\Delta {p}_{\text{cyt}}=\frac{{K}_{\text{shell}}}{a}\left\{{\left(\frac{a}{{a}_{0}}\right)}^{2}-1\right\}\left\{3{\left(\frac{a}{{a}_{0}}\right)}^{2}-1\right\}$$

Equation 6 represents an elastic constitutive equation for the cytoskeletal meshwork consisting of *N*_fiber_ elastic fibers or filaments anchored *at both ends* to the bilayer:

6 $$\Delta {p}_{\text{cyt}}=\frac{{N}_{\mathrm{fiber}}}{4\pi }{k}_{\mathrm{fiber}}{\left(\frac{{l}_{0}}{{a}_{0}}\right)}^{2}\frac{{a}_{0}}{{a}^{2}}(\frac{a}{{a}_{0}}-1)$$

where symbols *k*_fiber_ and *l*_0_ denote, respectively, the spring constant and the length of a fully contracted filament. The cell radii *a* and *a*_0_, correspond, respectively, to an expanded/stretched and a fully relaxed/contracted cytoskeleton. Equation 6 holds irrespective of whether interconnected fibers form a regular continuous meshwork (*e.g.*, spectrin cytoskeleton) or separate fibers are almost homogeneously distributed but randomly oriented over the inner surface of the RBC membrane, *e.g.*, NM2 filaments (Smith eth al., 2018). Detailed derivations of Eqs. 5 and 6 are given in the Supplement.

## Results

### RBC morphology and volume changes during electrohemolysis

**Fig. 1:**
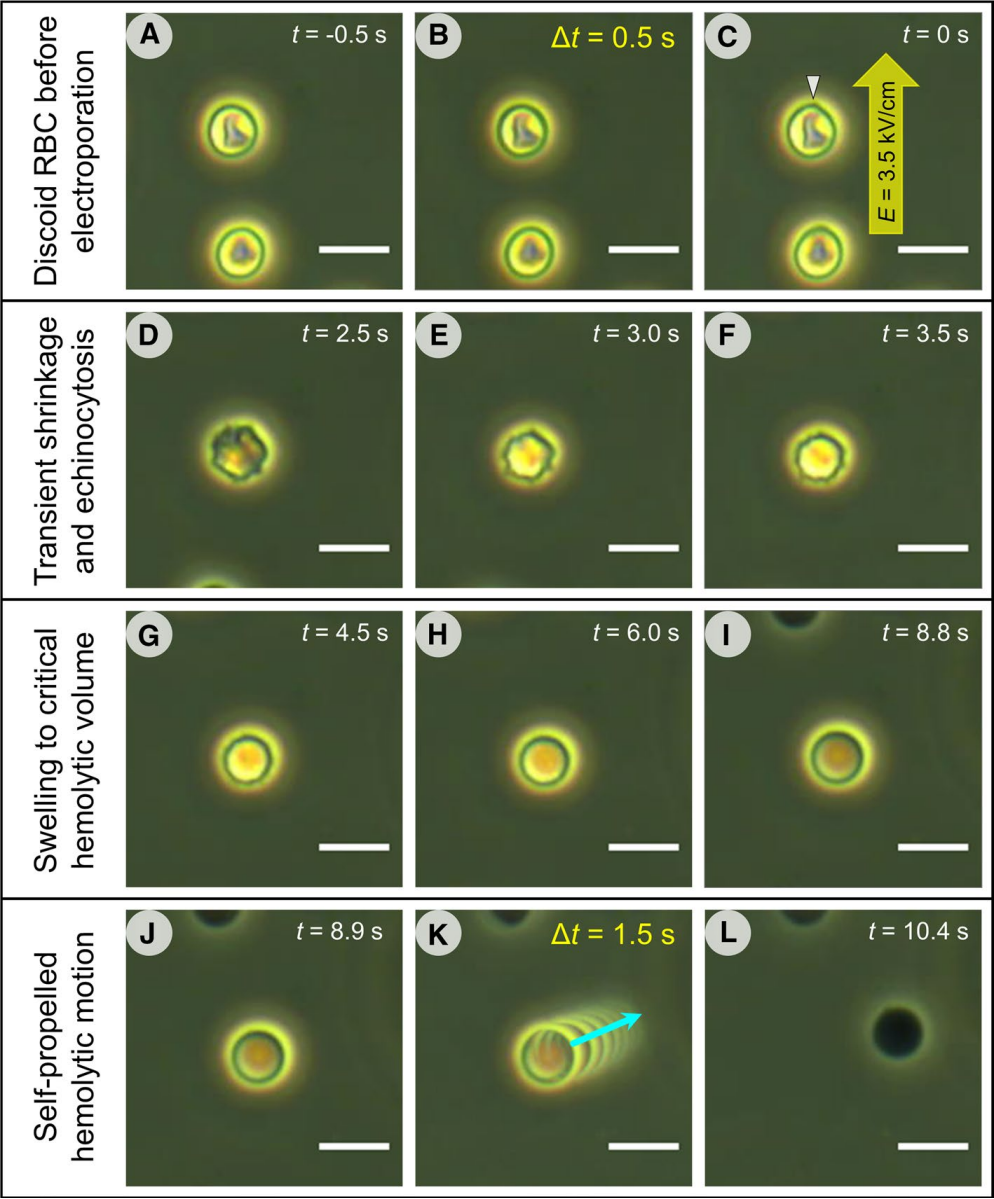
Electroporation-induced morphological changes of RBCs. **A** A normal discoid RBC, with a central dimple area ~ 1 s before electropulsing in isotonic sorbitol solution. **B** Superposition of several frames (Δ*t* = 0.5 s) indicates little, if any, cell displacement and shape changes before electropulsing. **C** Electropulsing causes a tip-like membrane protrusion (arrowhead) towards the cathode. **D-F** The electroporated cell first undergoes transient shrinkage and echinocytosis. Thereafter the cell swells (**G-I**) and converts to a spherocyte until reaching the critical hemolytic volume (**J**). The onset of hemolysis coincides with a sudden self-propelled linear motion of the cell (**K**, arrow). During hemolysis lasting ~ 1.5 s the erythrocyte gradually became less refractile due to the loss of Hb-rich cytoplasm, until finally it forms a Hb-depleted ghost (**L**). Scale bars: 10 µm

Figure 1 reveals the three morphological stages of an RBC undergoing electrohemolysis. During the first stage, the electroporated RBC (Fig. 1C) slightly shrank, lost its discoid shape, and converted to a spiculated echinocyte (Fig. 1D-F). Since the formation of echinocytes is commonly associated with ATP depletion (Lim et al., 2002; Braunmüller et al., 2012), the transient echinocytosis of electroporated RBCs is indicative of the ATP loss, e.g., via its leak-out through electropores. During the second stage, osmotic cell swelling caused gradual disappearance of spicules and formation of a smooth spherocyte *(*Fig. 1I-J). The cell swelled until the critical hemolytic volume *V*_crit_ of ~ 160 fL (Lim et al., 2008) is reached (Fig. 1J). The onset of hemolysis coincided with a sudden self-propelled cell motion (*K*), which can only result from a jet-like cytosol expulsion. At the end of the hemolytic motion, the cell transformed into a Hb-depleted ghost (Fig. 1L).

Interestingly, the series of shape transformations during electrohemolysis, *i.e.* echinocytosis, swelling, spherocytosis and ghost formation (Fig. 1), fully matches those reported for the RBC damage during storage (Putter and Seghatchian, 2017). The only difference is that electroporation induces these morphological changes within few seconds, whereas the RBC storage lesions progress much more slowly over several weeks (Putter and Seghatchian, 2017; Geekiyanage et al., 2020; Karandeniya et al., 2022). The following sections analyze the mechanism of the self-propelled RBC motion using image-based cell tracking and cell velocimetry.

### Velocimetry of the self-propelled motion of electroporated RBCs

**Fig. 2:**
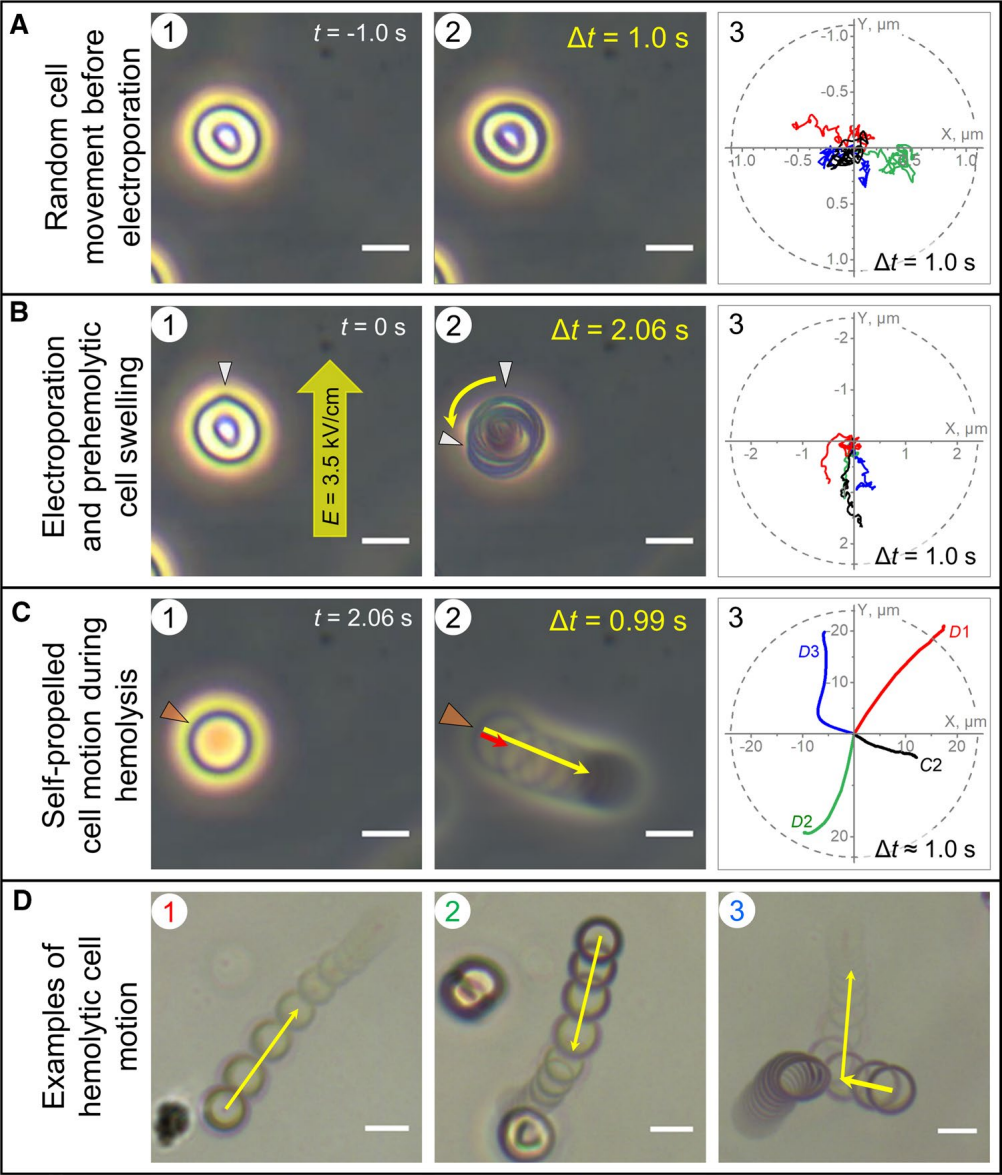
RBC motion before and after electroporation in isotonic sorbitol. **A** shows a discoid RBC (*A*1) and an overlay of 10 video frames (*A*2) taken over a time interval Δ*t* = 1 s before electropulsing. Cell tracking (*A*3) reveals random low-amplitude movement of the depicted (black line) and other freely suspended RBCs (colored lines) prior to electroporation. The cell is exposed to a supracritical DC pulse (arrow in *B*1). Electropulsing caused a transient tip-like membrane protrusion (arrowhead in *B*1) towards the cathode, corresponding to the largest of the electropores (*i.e.* prehemolytic hole). The electroporated cell displays slow (~ 1–2 µm/s) prehemolytic motion towards the anode and rotation (arched arrow in the overlay *B*2). The cell trajectory is illustrated by the black line in *B*3 along with trajectories of three other cells. White arrowheads in *B*2 indicate the initial and final locations of the prehemolytic hole, respectively, after electroporation and just before the onset of hemolysis. During the prehemolytic motion, the cell converted to a spherocyte (*C*1). **C** illustrates the actual hemolysis starting after the cell has swollen to its critical hemolytic volume (*C*1). Hemolysis was accompanied by fast cell motion consistent with a propulsion by cytosol ejection through the hemolytic hole acting as jet nozzle (orange arrowhead). The cell accelerated to a peak velocity of ~ 35 µm/s within the first 100 ms (red arrow in C2) and travelled a distance of ~ 12 µm for ~ 1 s (yellow arrow). **D** shows examples of hemolytic motion, including a rebounding collision of a hemolyzing cell (D3, cell on the right) with a slowly drifting spherocyte. During hemolysis (*C*_3_), the cells travelled much longer distances than during the pre-pulse (*A*_3_) and prehemolytic stages (*B*_3_). Cell contraction during hemolysis is evident from the reduction of the projected cell area clearly seen in images *D*1*-D*3. The trajectories of RBCs shown in *C*2 and *D*1*-D*3 are indicated by the corresponding symbols in graph C3. Scale bars: 5 µm. (Also *see* Supplemental Video S2)

We found that before electroporation (Fig. 2A), freely suspended cells displayed random, low-amplitude (< 0.5 µm/s) fluctuations about their mean positions (Fig. 2 A3). In contrast, electroporated cells not only swelled but also slowly displaced with velocities of 1–2 µm/s mainly towards the anodic side of the poration chamber (Fig. 2 B3). An anode-directed self-propelled motion can only arise from the propulsive force of a liquid jet of cytosol (except for hemoglobin) expelled through a jet nozzle, which apparently is the largest electropore (hereafter referred to as the prehemolytic hole), generated at the cell pole facing the cathode. Asymmetric electroporation of cells by direct-current (DC) electric pulses is well-known in the literature (Kinosita et al., 1988). When performed in low-conductive media, DC electroporation gives rise to a larger pore size at the cell pole facing the cathode (Tekle et al., 1994), which is fully consistent with our results obtained in low conductive (~ 100 µS/cm) sorbitol-substituted solutions. Finally, the occurrence of cytosolic leak-out jets is also indicative of a significant intracellular overpressure (Δ*p*_cyt_) during the prehemolytic phase.

Despite the cytosol leak-out (except for Hb) through the prehemolytic hole, electroporated RBCs continued swelling, which indicates that the osmotic water influx prevailed over the convective cytosol leak-out (see Osmo-elastic model in the Supplement). Upon swelling to the critical volume (Fig. 2 C1), RBCs abruptly started to hemolyze and to move in a self-propelled manner, travelling distances of up to 25–30 µm during ~ 1 s of hemolysis (Fig. 2 C3). The mean initial peak velocity *v*_max_ was ~ 35 µm/s (*see* below). About 5% of cells showed *v*_max_ of 60–90 µm/s (Fig. 2 D1). Most hemolyzing RBCs moved along linear trajectories (Fig. 2 C2, 2D1, 2D2). Occasionally, collisions with other cells altered the trajectory (Fig. 2 D3). Colliding cells were discarded from further velocimetric analysis.

During hemolytic motion, RBCs gradually decreased in size (Fig. 2D) and converted to Hb-depleted ghosts with a mean radius of ~ 2.61 ± 0.23 µm (Supplementary Fig. S3) corresponding to a volume of ~ 75 µm^3^ (= 75 fL). Given the critical hemolytic volume of ~ 160 fL (Fig. 2 C1), RBCs expelled ~ 55% of their cytosol during ~ 1 s of hemolysis. Interestingly, the mean ghost volume found here is similar to that of echinocytes (Chen et al., 2002), suggesting an elastic behavior of the RBC membrane during echinocyte-spherocyte-ghost transformation.

The only explanation for the fast self-propelled cell motion during hemolysis (Figs. 1–2) is the propulsive force exerted on the cell by a liquid jet of the pressurized cytosol ejected with large momentum. Moreover, the linear trajectories (Fig. 1 C3) of the hemolytic cell motion suggest that the cytosolic jet is ejected radially through a single membrane pore, *i.e.*, the hemolytic hole acting as a “jet nozzle”, while RBCs undergo contraction by expelling the cytosol, including Hb and other solutes. Cell size reduction during hemolysis is evident in Fig. 2 D1-D3.

**Fig. 3:**
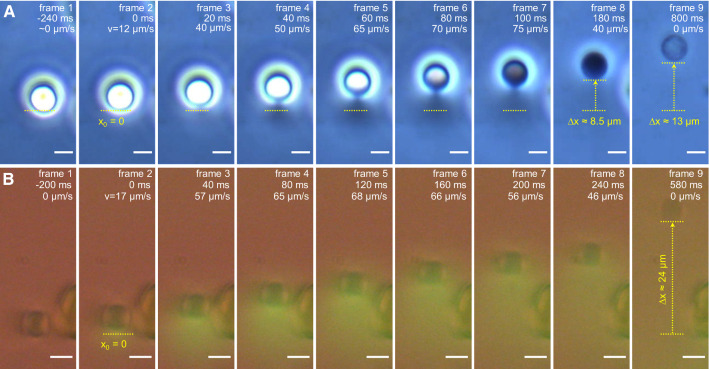
Emission of hemoglobin- (Hb) and carboxyfluorescein- (CF) jets from hemolyzing RBCs visualized, respectively, by phase-contrast (panel *A*) and fluorescence microscopy (*B*). The second image in each panel depicts the onset of hemolytic cell motion (*time* = 0 ms). Time stamps denote the time after the onset of hemolysis and the instantaneous cell velocity. **A** cell acceleration coincides with the emission of a cloud of refractile material (Hb-jet) in the direction opposite to cell motion. During the first 180 ms (frames 2–8), while the Hb-jet was clearly visible, the cell accelerated to a velocity of up to ~ 75 µm/s (frame 7) and travelled a distance of ~ 8.5 µm (frame 8). Thereafter the Hb jet was practically invisible, but the cell kept moving slower, travelled a distance of ~ 4.5 µm, came to a halt and converted to a ghost (frame 9). **B** combined fluorescence- and transmitted-light micrographs of an RBC loaded with CF prior to electroporation and emitting a green CF-jet during hemolysis. Scale bars: 5 µm

In line with the above reasoning, adjusting the phase-contrast microscope settings and the use of a fluorescence dye enabled the visualization of the cytosol jets emitted from RBCs during hemolytic motion (Fig. 3 and Supplementary movie S3). The concurrence of cytosol jets with cell acceleration provides clear-cut evidence that the self-propelled RBC motion is driven by the propulsive force generated by ejection of the pressurized cytosol.

### Inhibition of hemolytic cell motion by ATP and blebbistatin

Since cytosolic overpressure can only be generated by tension/contraction of the RBC membrane, we next analyzed the impacts of two known modulators of the RBC membrane elasticity, including ATP and blebbistatin (Braunmüller et al., 2012; Smith et al., 2018), on the hemolytic cell motion. ATP is well known to strongly modify the mechanical properties of RBCs, as demonstrated by the stiffening of the RBC membrane upon glucose/ATP deprivation and during storage (Betz et al., 2009; Turlier et al., 2016; Xu et al., 2018). As mentioned above, depletion of the cytosolic ATP in electroporated RBCs is highly likely to occur in an ATP-free poration buffer. Therefore, extracellular ATP should prevent cytosolic ATP depletion.

**Fig. 4:**
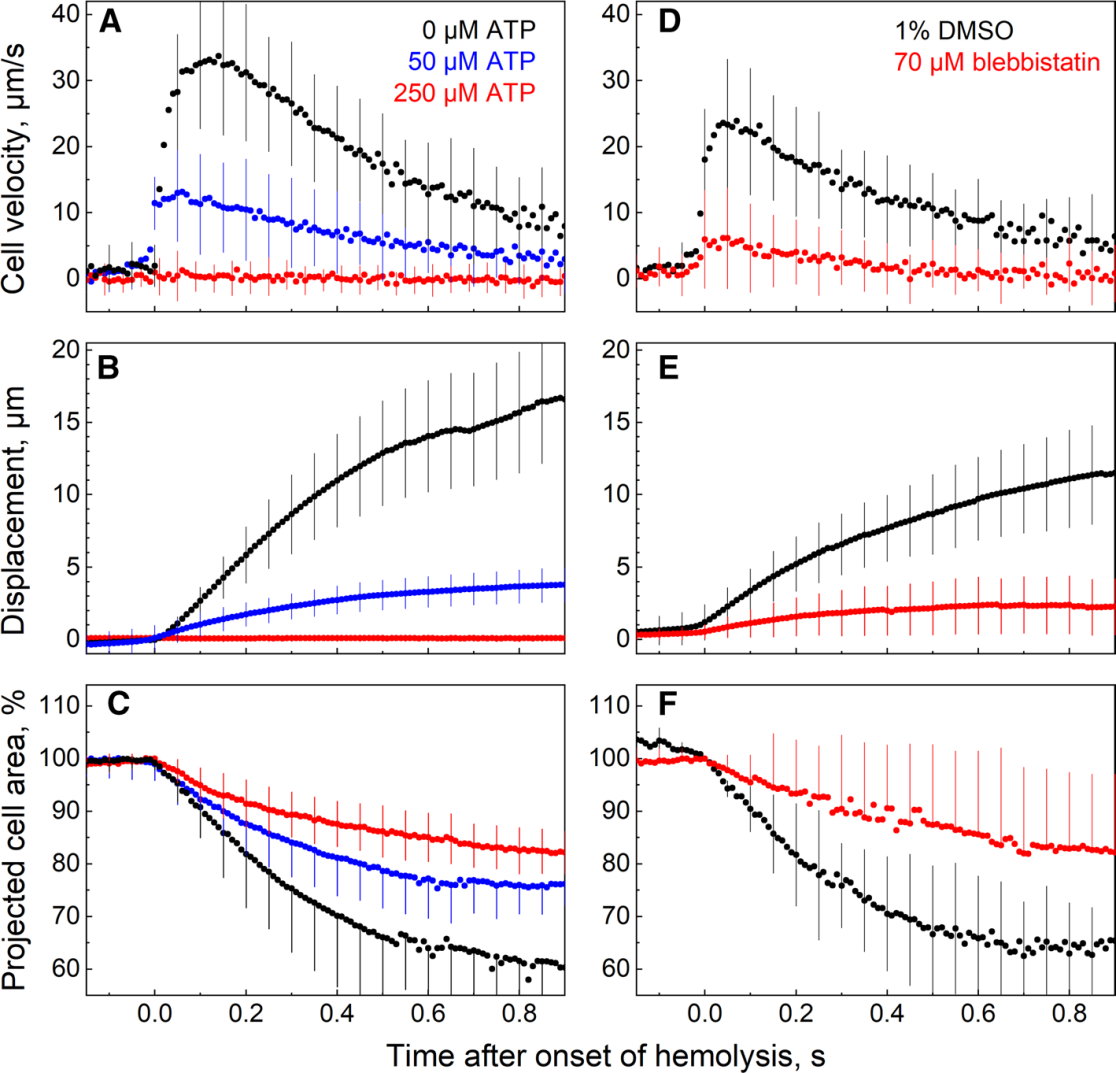
Impact of ATP and blebbistatin on the RBC velocity, displacement and projected area. Each symbol represents the mean value (± SD) of 40–50 cells. For each cell, the origin of the time axis (*t* = 0) was set to the onset of hemolysis corresponding to the maximum cell acceleration. **A-C** Hemolysis of control cells (black symbols) started suddenly with a fast cell acceleration to the peak velocities of ~ 35 µm/s. **B** During hemolysis, control RBCs travelled a mean total distance of ~ 15 µm. Cell acceleration coincided with the onset of a rapid decrease in the projected cell area (**C**), which evinces cell contraction and volume decrease. After reaching the maximum velocity (*t *≈ 150 ms), RBCs gradually decelerated (***A***) and decreased in size (**C**). Extracellular 50 µM ATP (*blue symbols*) significantly reduced the peak velocity, whereas 250 µM ATP (*red symbols*) fully abolished RBC motion (*A, B*) and reduced cell contraction (*C*) during hemolysis. **D–F** Blebbistatin (70 µM) (*red symbols*) reduces ~ 5–sixfold the peak velocity and displacement as compared to DMSO-treated controls (*black symbols*). The extent of hemolytic cell contraction was also decreased by blebbistatin (**F**). For statistical comparison of the peak velocities of ATP- and blebbistatin-treated cells with the respective controls see Supplemental Fig. S4

Surprisingly, we found that supplementing the poration buffer with 50 µM ATP strongly reduced the peak velocity of the RBCs, whereas 250 µM ATP fully abolished any detectable cell motion (Fig. 4AB). The ATP-mediated inhibition of RBC motion resulted obviously from a decreased expulsion of the cytosol, which is evident from the rates and extents of cell contraction in the presence of ATP (Fig. 4C). Our results indicate that cytosolic ATP depletion is a prerequisite for hemolytic cell-contraction and -motion.

ATP depletion is also known to be associated with the rigor configuration of the actomyosin complex (Finer et al., 1994; Brito and Sousa, 2020). Since the non-muscle myosin 2A (NM2A) is known to interact with the actin junctions of the RBC cytoskeleton (Smith et al., 2018), we further analyzed the involvement of NM2A in the hemolytic RBC motion using the membrane permeable myosin inhibitor blebbistatin. Blebbistatin selectively inhibits the ATP-dependent *motor activity* of NM2 by dissociating myosin from actin (Rauscher et al., 2018). It also decreases the number of NM2A filaments associated with the RBC cytoskeleton (Smith et al., 2018). We found that 70 µM blebbistatin reduced ~ 5–sixfold the peak velocity and displacement during hemolytic cell motion, as compared to DMSO treated control (Fig. 4D-4F). Interestingly, electroporated RBCs treated with ATP or blebbistatin do not undergo transient echinocytosis prior to hemolysis (Supplemental video S4).

Taken together, the data presented in Fig. 4 suggest that the hemolytic cell motion involves both the ATP-dependent NM2 motor activity and the ATP-depleted actomyosin rigor configuration. Yet since ATP also modulates the RBC elasticity via the coupling/decoupling of the spectrin-actin network to the bilayer (Gov, 2007), we first explored the possible involvement of the bilayer and the spectrin cytoskeleton in the hemolytic cell motion.

### Comparison between experiments and the elastic models for the bilayer, spectrin cytoskeleton and myosin filaments

To assess to which extent the bilayer and the cytoskeleton contribute to the propulsive behavior of hemolyzing RBCs we analyzed our experimental data with the minimal elastic model, according to which the self-propelled hemolytic motion is driven by ejection of the pressurized cytosol through a hemolytic hole (Hb-jet). The cytosolic overpressure is generated by the contraction of either the membrane bilayer or the cytoskeleton, which are integrated into the model via their individual elastic constitutive equations. Using a more general osmo-elastic model (*see* Supplement), we show that the osmotic water influx through the membrane has little effect on the hemolytic cell motion. Therefore, the osmotic flux during hemolysis is neglected in the minimal model. The model assumes spherical cell geometry and comprises Eqs. 1–6 given in the Materials and Methods section.

It is well known that the elasticity modulus of the RBC bilayer, *K*_BL_ ≈ 0.5 N/m, (Mohandas and Evans, 1994) exceeds by ~ five orders of magnitude that of the cytoskeleton (*K*_CS_ ≈ 10 µN/m) reported for normal discoid RBCs (Engelhardt and Sackmann, 1988; Dao et al., 2006). Accordingly, contribution of the cytoskeleton contraction to cytosolic pressure generation during hemolytic motion is negligible as compared to that of the RBC bilayer.

However, the nearly inextensible lipid bilayer can only withstand a maximum area expansion *ΔA*_crit_/*A*_0_ of ~ 3–4% before it releases tension via hemolytic hole formation (Evans et al., 1976; Boal, 2012). Accordingly, a tension-driven 3–4% bilayer area contraction to the tensionless state would only expel a small portion (~ 4.5–6%) of the cytosol, which clearly contradicts the observed ~ 55% cytosol expulsion upon cell-to-ghost transition during hemolytic motion. The same is true for the bilayer-anchored spectrin cytoskeleton, whose area equals that of the bilayer. Therefore, a priori elastic contraction of the bilayer and/or the spectrin cytoskeleton cannot generate small Hb-depleted ghosts observed in experiments (Fig. S3).

**Fig. 5:**
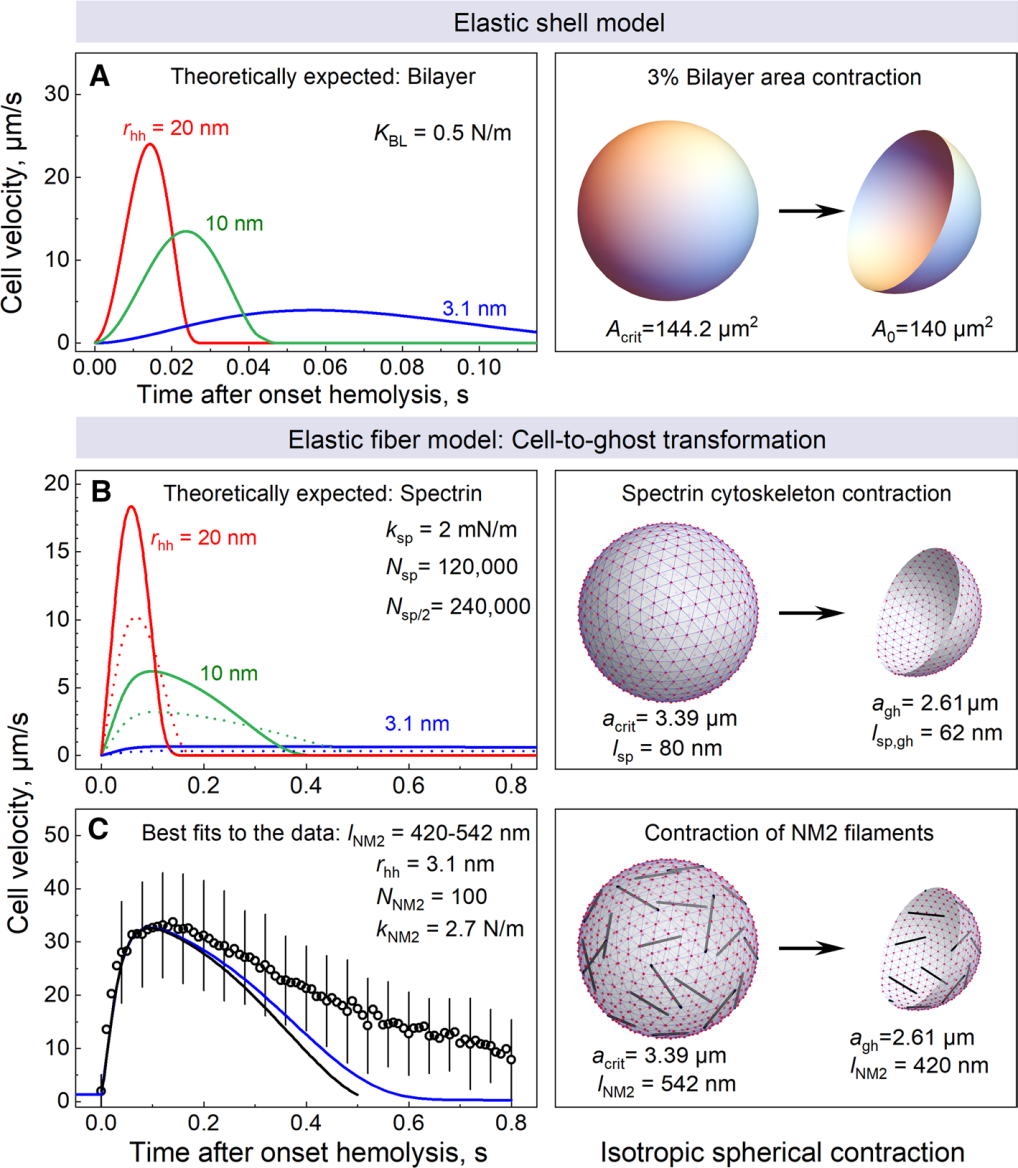
Neither the bilayer (**A**) nor the spectrin cytoskeleton (**B**) account for the hemolytic RBC motion as shown by comparison of the experimental results with the elastic-shell and -fiber models. The curves in **A** and **B** were calculated for hemolytic hole radii *r*_hole_ of 3.1, 10 and 20 nm, assuming *τ*_hole_ = 20 ms (Eq. 3). The fitted curve in **C** was calculated for the *r*_hole_ = *r*_Hb_ = 3.1 nm. In **A**, cell velocity curves are shown for an RBC motion driven solely by bilayer contraction. Accordingly, the initial tensed and the final relaxed bilayer areas were *A*_crit_ = 1.03 × *A*_0_ = 144.2 µm^2^ and *A*_0_ = 140 and, respectively, and bilayer elasticity *K*_BL_ = 0.5 N/m. In **B**, cell velocity curves were generated with the elastic fiber parameters consistent with the spectrin cytoskeleton (stiffness *k*_sp_ = 2 mN/m) and the observed cell-to-ghost transformation. The initial tensed cell and final relaxed ghost radii were set to 3.39 and 2.61 µm, respectively. Continuous lines in **B** were calculated for spectrin fiber lengths of *l*_sp_ = 80 nm and *l*_sp,gh_ = 62 nm assuming 120,000 actin-to-actin anchored spectrin filaments. Dashed lines were calculated for fiber lengths of *l* = 40 and *l*_sp,gh_ = 31 nm assuming 240,000 actin-to-ankyrin connected half-spectrin filaments. The black curve in **C** is the best fit of the elastic-fiber model to the data using the reported length of NM2 filaments of 420 nm (Smith et al., 2018), yielding 100 filaments with a spring constant *k*_NM2_ of 2.7 N/m. The blue curve was calculated with the general osmo-elastic model (For detail see text and the Supplement Fig. S9.)

In line with the above reasoning, curves generated with the elastic shell model (Eqs. 1–5) using bilayer parameters (*K*_shell_ = *K*_BL_ = 0.5 N/m and 3% area contraction) do not match the experimental velocity data, i.e., the propulsive force provided by the bilayer contraction would persist only for ~ 20–100 ms (Fig. 5A), which is far too short to maintain the observed 1 s of hemolytic cell motion (Fig. 3AB). Consistent with our conclusion that the elastic contraction of RBC membrane bilayer cannot be responsible for the hemolytic motion, no detectable displacement was reported for giant unilamellar phospholipid vesicles upon convective leak-out of the inner solution through a membrane pore (Brochard-Wyart et al., 2000; Chabanon et al., 2007).

Next we analyzed the RBC motion data with the elastic fiber model (Eq. 6) allowing for the molecular structure and elasticity of the spectrin cytoskeleton, along with the observed RBC contraction from *a*_crit_ = 3.39 µm to *a*_ghost_ = a_0_ = 2.61 µm, during cell-to-ghost transformation. The number of fibers *N*_fiber_ in Eq. 6 was set to the number of spectrin filaments, *N*_fiber_ = *N*_sp_ = 120,000 per RBC (Lux, 2016). Spectrin filaments form a triangular network via interconnections to 40,000 actin-based junctions (nodes) anchored in the bilayer, as illustrated by gray lines and red circles in the RHS cartoon in Fig. 5B. In normal RBCs, the length of spectrin filaments *l*_sp_ equals the distance between the actin nodes they connect *l*_sp_ ≈ 80 nm (Pan et al., 2018). In order to bring about an isotropic spherical cell contraction, the length of spectrin filaments would have to decrease (via unknown mechanism) from *l*_sp_ = 80 nm to *l*_sp,gh_ = 58 nm, because of the linear relationship between the sphere chord length and the sphere radius (*l*_sp,gh_/l_sp_ = a_gh_/a_crit_). To account for spectrin-to-ankyrin connections, we also tested the elastic fiber model by assuming 240,000 actin-to-ankyrin connected half-spectrin filaments of length *l*_sp/2_ = 40 nm (dashed lines in Fig. 5B).

In most molecular models of RBC elasticity, flexible spectrin filaments behave, for small deformations, as Hookean entropic springs with a spring constant *k*_sp_ of few µN/m (Boal, 2012; Zhang and Brown, 2008). At larger extensions, spectrin filaments behave nonlinearly, exhibiting strain stiffening and unfolding (Gov, 2007). Accordingly, whole cell measurements on discoid RBCs commonly yield a very low value of ~ 10 µN/m for the physiological elasticity of the RBC cytoskeleton (Turlier et al., 2016), whereas, in isolated spectrin molecules, single spectrin repeats unfold with a spring constant *k*_sp_ of ~ 1 mN/m (Rief et al., 1999; Randles et al., 2007). Given that a spectrin filament is formed by two laterally associated αβ heterodimers (Lux, 2016), its spring constant is expected to be twice that of a single spectrin molecule, i.e., ~ 2 mN/m, which by far exceeds the physiological elasticity of the RBC cytoskeleton (~ 10 µN/m).

As seen in Fig. 5B, curves generated with the elastic fiber model using spectrin parameters do not match the experimental velocity data (symbols in Fig. 5C). Even by assuming a physiologically unlikely *k*_sp_ = 2 mN/m for spectrin filaments, a noticeable propulsive force they provide would persist only for 0.1–0.4 s or be negligible (Fig. 5B).

Taken together, in agreement with the above-mentioned inextensibility of the RBC membrane (i.e., maximum ~ 3% area strain), our theoretical analyses (Fig. 5AB) show that neither the bilayer nor the spectrin filaments can be responsible for the propulsive behavior of hemolyzing RBCs driven by expulsion of ~ 55% of the cytosol within ~ 1 s observed in our experiments (Fig. 5C). We are therefore left with only one possible mechanism for the Hb-jet driven cell motion, which involves the RBC myosin filaments composed of the non-muscle myosin 2A (NM2A) (Smith et al., 2018).

Unlike the abundant spectrin filaments (~ 120,000 per RBC), the ~ 6200 NM2A molecules per RBC (Fowler et al., 1985; Wong et al., 1985) can form up to ~ 200 filaments, although only ~ 70 were visualized (Smith et al., 2018). The bipolar NM2A filament consists of ~ 30 myosins with both filament ends each presenting ~ 30 motor domains at their globular heads (Costa and Sousa, 2020). While the 80-nm long spectrin filaments connect adjacent actin nodes, the much longer NM2A filaments can bind with their ends to and bridge actin nodes spaced by ~ 420 nm and larger (Smith et al. 2018). But unlike spectrin, the low abundant NM2 filaments are unable to create a regular continuous network and are distributed randomly over the cytosolic membrane surface, thus forming a discontinuous scaffold attached to the RBC membrane, as illustrated schematically in Fig. 5C. Moreover, myosin motorheads are tightly locked to actins only in the ATP-free rigor configuration but not during the ATP dependent motor activity (Brito and Sousa, 2020).

To test whether the elastic contraction of NM2 filaments can bring about the hemolytic cell motion, we applied the elastic fiber model to the cell motion data (curve in Fig. 5C) by using the length of a relaxed NM2 filament *l*_NM2,gh_ = 420 nm (Smith et al., 2018). The length of a fully stretched NM2 filament was calculated as *l*_NM2,crit_ = *l*_NM2,gh_ × *a*_crit_/*a*_gh_ ≈ 542 nm, where *a*_crit_ = 3.39 µm and *a*_ghost_ = 2.61 µm. By varying the number of NM2 filaments between the reported 70 (Smith et al. 2018) and the expected ~ 200 (Fowler et al., 1985; Wong et al., 1985), we found the range of 1.4–3.9 N/m for the effective spring constant *k*_NM2_ of a single filament, with a range mid-point *k*_NM2_ of ~ 2.7 N/m (Fig. 5C). Given that a bipolar NM2 filament consists of 30 laterally associated NM2 monomers attached to each other by their tail domains (Costa and Sousa, 2020), the stretching stiffness of a single NM2 protein can be estimated as *k*_NM2_/30 =  ~ 2.7/30 N/m ≈ 90 mN/m. This value matches very well the stretching stiffness of 60–80 mN/m reported for the NM2 tail domain (Adamovic et al., 2008). Taken together, using the minimal elastic-fiber model we showed that despite their low abundance the long and stiff NM2 filaments can provide the driving force for hemolytic cell motion via generation of the cytosolic overpressure of ~ 35 kPa at the onset of hemolysis (supplemental Fig. S8D).

The inhibitory effect of ATP on the hemolytic cell motion (Fig. 3A) and the appearance of ATP-deprived echinocytes (Fig. 1) strongly suggest that vanishing of the NM2 motor activity upon ATP depletion is necessary for hemolytic cell motion. Thus, the Hb-jet expulsion apparently involves NM2 filaments tightly locked to actin in a rigor configuration (Brito and Sousa, 2020; Finer et al., 1994), as we argue in the Discussion section. We also propose a putative mechanism of elastic stretching of NM2 filaments during swelling of echinocytes, leading to the generation and maintaining of the cytosolic overpressure during prehemolytic cell swelling and the ensuing hemolytic motion.

## Discussion

In this study, we explored the biomechanics of rapid Hb expulsion during hemolysis (Videos S1 and S2). We found that hemolysis was often preceded by transient echinocytosis followed by cell swelling to the critical hemolytic volume (Fig. 1). The onset of hemolysis coincided with sudden cell acceleration (Fig. 2), driven by jet-like expulsion of the pressurized Hb-rich cytosol (Fig. 3). The hemolytic cell contraction and motion was strongly inhibited by blebbistatin and ATP (Fig. 4).

**Fig. 6:**
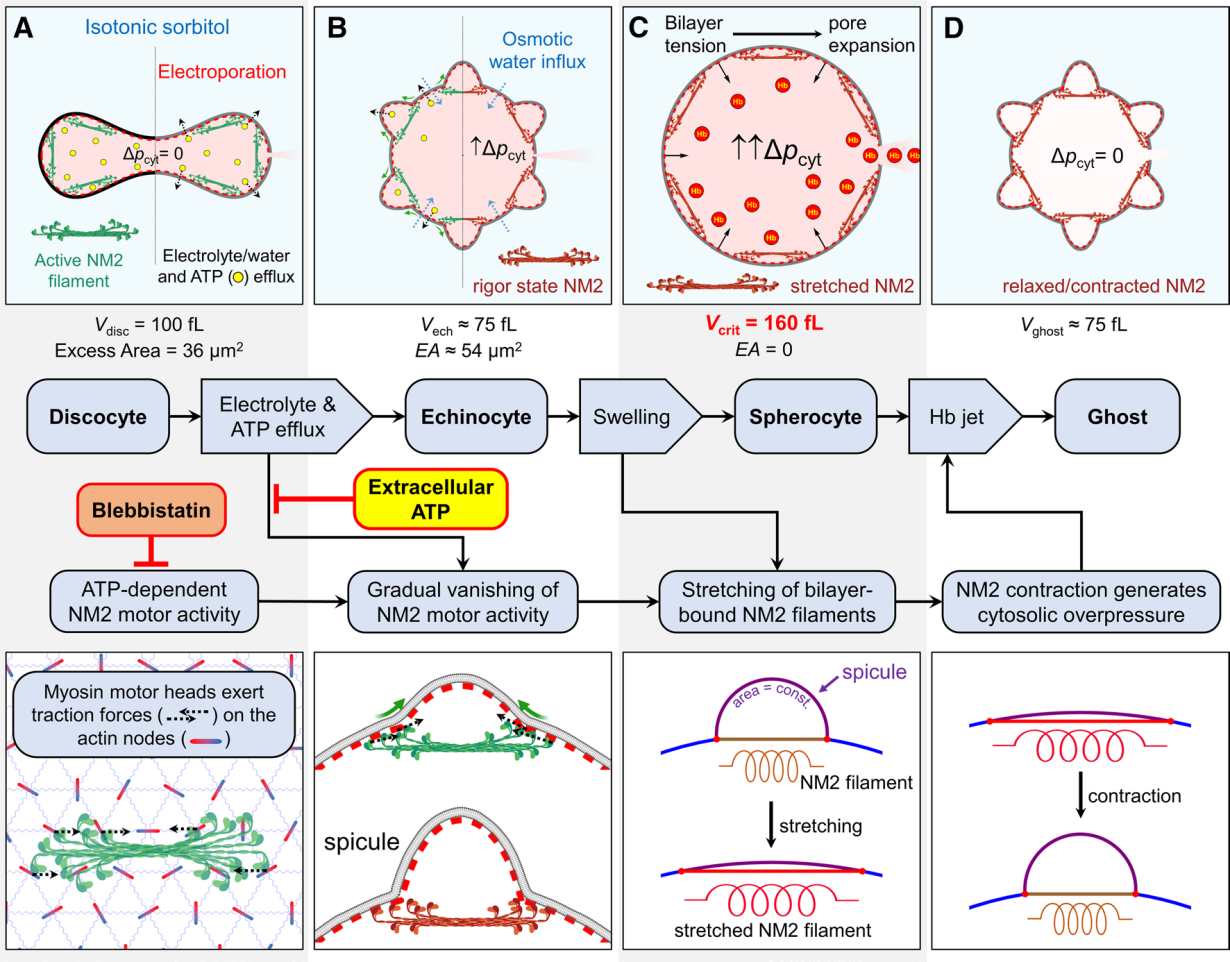
Biomechanics of rapid hemoglobin expulsion driven by interactions of bipolar NM2 filaments with the actin filaments of the RBC cytoskeleton. **A** Electroporation of a discocyte renders its membrane permeable to small solutes, including K^+^, Cl^−^, ATP and sorbitol. **B** The rapid ion efflux and the leak-out of ATP through electropores leads, respectively, to transient cell shrinkage and gradual vanishing of the NM2 motor activity giving rise to spicule formation and echinocytosis. The ensuing cell swelling due to sorbitol influx and colloid-osmotic pressure causes spherocytosis with flattening of spicules, accompanied by stretching of NM2 filaments bound in a chord-like manner to the bilayer at the spicule base. The stretching-induced tensile forces of NM2 filaments are balanced by the elastic bilayer tension (σ_BL_), which in turn generates intracellular pressure (∆*p*_cyt_, RHS of image *B*) in accordance with the Laplace equation: ∆*p*_cyt_ = σ_BL_/*a*_cell_, where *a*_cell_ is the cell radius. The presence of a substantial ∆*p*_cyt_ during swelling of echinocytes is evinced by their slow prehemolytic motion (Fig. 2 B3). During this phase, the bilayer tension σ_BL_ was apparently not sufficient to induce significant expansion of electropores. **C** First, upon swelling to the critical hemolytic volume (~ 160 fL), the strain-induced bilayer tension became large enough to initiate the expansion of the largest electropore to form a hemolytic hole. Once the hole reaches the radius of Hb, expulsion of the Hb-rich cytosol occurs driven mainly by the contractile force of stretched NM2 filaments, resulting in hemolytic cell motion and formation of Hb-depleted ghosts (**D**). (The figure was partially created with BioRender.com.)

The only plausible mechanism for contraction of hemolyzing RBCs appears to involve both the ATP-dependent motor activity and the ATP-depleted rigor state of NM2 filaments interacting with the membrane-bound short actin filaments. As already mentioned above, each end of a 420-nm long NM2 filament presents a bundle of 30 globular motorheads (Fig. 6A) (Smith et al., 2018). The motorheads at both filament ends can spread out over more than 100 nm (Billington et al., 2013). Therefore, each NM2 filament end can simultaneously engage ~ 3–4 actin junctions (Fig. 6A, bottom graph) each spaced ~ 80 nm apart (Lux, 2016; Pan et al., 2018). The active NM2 motor heads (green color in Fig. 6) will exert traction forces on the actins (dashed arrows in Fig. 6AB bottom graph) thus pulling together the two groups of engaged actin nodes anchored in the bilayer.

In the presence of a large excess of membrane area in discoid RBCs (Fig. 6) exhibiting a very small membrane bending modulus *k*_b_ of 2 × 10^–19^ N/m (Lim et al. 2008), the two oppositely directed, transient traction forces exerted by an NM2 filament on actins tangentially to the bilayer (Picart et al., 2000) will cause local membrane folding by producing a convex membrane protrusion above the underlying NM2 filaments (Fig. 6B bottom grap). Moreover, the height of the protrusion will certainly alternate as a result of the ATP-dependent NM2 motor activity, including the motor head sliding on, detachment from and engagement of actin filaments of variable distance and orientation.

As a side note, it has not escaped our notice that the traction forces exerted by NM2 on the RBC cytoskeleton (dashed arrows in Fig. 6B, bottom graph) may be involved in the RBC membrane fluctuation also known as flickering. At a sliding velocity of the motor head movement of ~ 300 nm/s along actins at a physiological ATP concentration of 1 mM (Heissler and Manstein, 2013), the traction force duration exerted by a single motor head would be ~ 120 ms, i.e., the time needed to travel over a 37-nm short actin filament. As a result, the traction forces on actins are likely to alternate on a timescale of ~ 100 ms, thus leading to fluctuations in the height of membrane protrusions. Recent studies of RBC flickering revealed local ATP-dependent mechanical forces of so far unknown molecular origin exerted on the cytoskeleton at a timescale of ~ 100 ms (Turlier et al., 2016; Rodríguez-García et al., 2015). Moreover, the low amplitude of the flickering forces of ~ 5 fN (Rodríguez-García et al., 2015) directed normal to the membrane is consistent with the much stronger myosin force of ~ 3–4 pN (Finer et al., 1994) exerted on actin tangentially to the membrane. Rodríguez-García et al. (2015) also report that ATP depletion converts most RBCs to rigid nonfluctuating echinocytes. Thus, in view of the similarity in their ATP dependence and force durations, we hypothesize that myosin motor activity is involved in the RBC membrane flickering. Likewise, Turlier and Betz (2019) have also suggested a possible role of NM2 in membrane flickering of eukaryotic cells more complex than RBCs.

In electroporated RBCs, the concentrations of cytosolic ions (K^+^, Cl^−^, etc.) and other small solutes including ATP_i_ rapidly decrease due to their efflux through electropores (Saulis and Saule, 2012). Whereas the ion efflux leads to a transient cell volume decrease (see Supplement “General osmo-elastic model”), ATP depletion inhibits the dissociation of myosin from actin thus decreasing the sliding velocity (Finer et al., 1994). As a result, the duration of the traction forces of NM2 on actins increases, which in turn promotes the formation of more durable convex membrane protrusions known as spicules of echinocytes (Fig. 6B, bottom graph). In line with the NM2 mechanism proposed here, spicules emerging at the initial stage of echinocytosis, i.e., when NM2 heads are still able to detach from and reattach to actin junctions, exhibit random movement over the RBC surface (Melzak et al., 2020). Since the RBC volume decreases during echinocytosis from 100 to ~ 75 fL (Chen et al., 2002), the large excess (~ 40%) of membrane area allows the formation of up to ~ 50–60 spicules per cell (Brecher and Bessis, 1972; Reinhart and Chien, 1986).

Upon complete ATP_i_ depletion, the myosin motorheads become tightly locked to actin junctions in the rigor configuration (Brito and Sousa, 2020). At this stage, according to our model, a bipolar NM2 filament bridges two groups of actin nodes located diametrically in the spicule base region (brown color Fig. 6B). Consistent with the NM2-based mechanism of spicule formation proposed here, the spicule diameters of ~ 500 nm measured from the electron microscopic image of an echinocyte (Brecher and Bessis, 1972) compare reasonably well with the NM2 filament length of ~ 420 nm (and larger) determined by fluorescence microscopy (Smith et al., 2018). In addition, a good agreement between the number of spicules per echinocyte of up to 50–60 (Brecher and Bessis, 1972; Reinhart and Chien, 1986) and the number of membrane-associated NM2 filaments in discocytes (~ 70) (Smith et al., 2018) lends further support for the involvement of NM2 in the RBC membrane spiculation. In agreement with this line of reasoning, we found that ATP and blebbistatin abolished (via different mechanisms) both the hemolytic cell motion (Fig. 3) and the transient echinocytosis (Video S4), by promoting the dissociation of the rigor actin-myosin complex (*i.e.,* by ATP, Fig. 6) or by preventing the binding of NM2 to actin (by blebbistatin).

According to the common bilayer-couple theory (Sheetz and Singer, 1974), formation of the echinocytotic spicules involves an area expansion of the outer bilayer leaflet relative to the inner one (Lim et al., 2002; Bernhardt et al., 2008). Among other reasons, an expansion of the outer leaflet can be caused by a conformational change in the cytoskeleton-anchoring transmembrane protein band 3 (Gimsa and Read, 1995; Gimsa, 1998; Betz et al. 2007), a significant portion of which is located in the actin junctions (Burton and Bruce, 2011; Lux, 2016). It is therefore conceivable that the mechanical forces exerted by NM2 filaments on the engaged actin junctions would alter the band 3 conformation thus triggering the formation of spicules.

For simplicity, a spicule can be viewed as a hemispherical membrane cap connected at its base to the rest of the cell body. The radius of the circular spicule base (*a*_spic_) equals half the length of a relaxed NM2 filament (*L*_NM2_ = 420 nm): *a*_spic_ = *L*_NM2_/2. As illustrated in the 2D cross-sectional view of the spicule (Fig. 5C bottom), the NM2 filament is connected in a chord-like manner to the actin junctions (red dots) located at the border between the spicule (purple) and the main cell membrane (blue). The spicule surface area (*A*_spic_) is equal to that of a hemisphere with a radius of curvature *a*_spic_ = 210 nm: ${A}_{\mathrm{spic}}=2\pi {a}_{spic}^{2}=\pi {L}_{\mathrm{NM}2}^{2}/2$. During the following swelling-mediated transition from echinocyte to spherocyte with a critical volume *V*_crit_ = 160 fL, the radius of spicule curvature approaches that of the main cell body, *i.e.* spherocyte: *a*_cell_ = 3.39 µm ($a_{{{\text{cell}}}} = \sqrt[3]{{\left( {3V_{{{\text{crit}}}} /4\pi } \right)}}$. Given that the bilayer is nearly inextensible (*i.e. A*_spic_ = const.), the increase in the radius of spicule curvature (from 0.21 to 3.39 µm) will result in the stretching of the underlying NM2 filament. The length of a stretched NM2 filament can be calculated as ${L}_{NM2}^{str}=2{a}_{\mathrm{cell}} \mathrm{sin}\left(\mathrm{arccos}\left(1-{\left({a}_{\mathrm{spic}}/{a}_{\mathrm{cell}}\right)}^{2}\right)\right)=590 \mathrm{nm}$ (Supplement Fig. S9), which agrees well with the length of ~ 542 nm used for fitting the elastic-fiber model to the cell velocity data in Fig. 4B.

It is well known that in sharp contrast to normal discoid RBCs, ATP-depleted echinocytes are strikingly rigid cells with an increased resistance to osmotic swelling and lysis (Dreher et al., 1978; Clark et al. 1981; Braunmüller et al. 2012). Our model explains the reported rigidity of echinocytes by the presence of a stiff scaffold of ~ 100 NM2 filaments attached via the actin nodes to the spiculated RBC membrane. The reported resistance to osmotic swelling is apparently due to the NM2-stretching associated cytosolic overpressure ∆*p*_cyt_, which opposes the osmotic water influx into electroporated RBCs (see Eq. S22 in the Supplement). Interestingly, rigid RBCs are also highly resistant to electrohemolysis (Mussauer et al., 1999).

The theoretical models used in this study yielded a similar peak value of ~ 35–40 kPa for the hydrostatic pressure in the cytosol ∆*p*_cyt_ at the onset of hemolytic motion (Supplementary Figs. S5 and S8). The cytosolic pressure increases during the cell swelling phase due to the stretching of NM2 filaments attached in the rigor state to the actin nodes (Fig. S8D). Moreover, once the critical hemolytic volume is reached, further swelling generates tension in the bilayer. As a result, the largest electropore expands to a hemolytic hole, which allows escape of Hb molecules (Fig. 6C, upper graphic). Consequently, contraction of the stretched NM2 filaments provides the driving force for the cytosol ejection, which leads to formation of Hb-depleted ghosts (Fig. 6D). During ~ 1 s of hemolysis, ~ 55% of the cytosol is ejected by contraction of osmotically stretched NM2 filaments. For comparison, releasing the same amount of Hb via passive diffusion through a hemolytic hole with a radius 3.1 nm would take up to ~ 1.5 h (*see* Supplement, Eq. S15), depending on the concentration-dependent diffusion coefficient of Hb (Adams and Fatt, 1967).

In conclusion, our study reveals an actomyosin-based mechanism of bulk Hb expulsion during hemolysis. This mechanism may be of importance in the splenic clearance of senescent RBCs, where hemolysis is a key event preceding the recognition and ghost degradation by red pulp macrophages (Klei et al., 2020). Since every second the human blood must be cleared of ~ 5 million rigid senescent RBCs (Thiagarajan et al., 2021), it is conceivable that their hemolysis in the spleen relies on a fast Hb-expulsion rather than on a slow diffusive Hb-efflux. Moreover, the results of this study may be of interest to researchers working in the field of blood storage and transfusion. Particularly, our conclusion that the rigor actomyosin is involved in echinocytosis and hemolysis might be relevant for the development of new protocols for extending the shelf-life of preserved blood by diminishing storage-associated RBC lesions.

## Supplementary Information

Below is the link to the electronic supplementary material.

Supplementary file1 (PDF 1264 KB)

Supplementary file2 (AVI 2726 KB)

Supplementary file3 (AVI 1130 KB)

Supplementary file4 (AVI 977 KB)

Supplementary file5 (AVI 8500 KB)
