# Supplementary material for: Contraction of the rigor actomyosin complex drives bulk hemoglobin expulsion from hemolyzing erythrocytes
Source: Biomech Model Mechanobiol. 2022 Nov 10;22(2):417–32. doi: 10.1007/s10237-022-01654-6 (PMC10097772; doi:10.1007/s10237-022-01654-6)
Supplement: Supplementary file 1 — Supplementary file1 (PDF 1264 KB) [file 10237_2022_1654_MOESM1_ESM.pdf]

## SUPPLEMENTARY INFORMATION FOR

# Contraction of the rigor actomyosin complex drives bulk hemoglobin expulsion from hemolyzing erythrocytes

Ryo Shirakashi,[1,§] Dmitri Sisario,[2,§] Danush Taban,[2] Tessa Korsa,[2,4] Sophia B. Wanner,[2] Julia Neubauer,[4] Cholpon S. Djuzenova,[3] Heiko Zimmermann,[4-6] and Vladimir L. Sukhorukov[2,*]

[1]Institute of Industrial Science, The University of Tokyo, Tokyo, Japan

[2]Department of Biotechnology & Biophysics, Biocenter, University of Würzburg, Würzburg, Germany

[3]Department of Radiation Oncology, University Hospital of Würzburg, Würzburg, Germany

[4]Fraunhofer Institute for Biomedical Engineering (IBMT), Sulzbach, Germany

[5]Department of Molecular and Cellular Biotechnology, Saarland University, Saarbrücken, Germany

[6]Faculty of Marine Science, Universidad Católica del Norte, Coquimbo, Chile

# 1. SUPPLEMENTARY METHODS AND RESULTS

## Electroporation chamber and electric pulses

Most RBCs exposed to isotonic sorbitol solution exhibited a biconcave, discoid shape (Fig. S1A and movie S1). Within few seconds after being transferred to the glass-bottomed electroporation chamber (see below), most cells floated close to the glass slide with a dimpled side facing the glass surface. With this orientation, the applied electric field is parallel to the disc planes of biconcave RBCs.

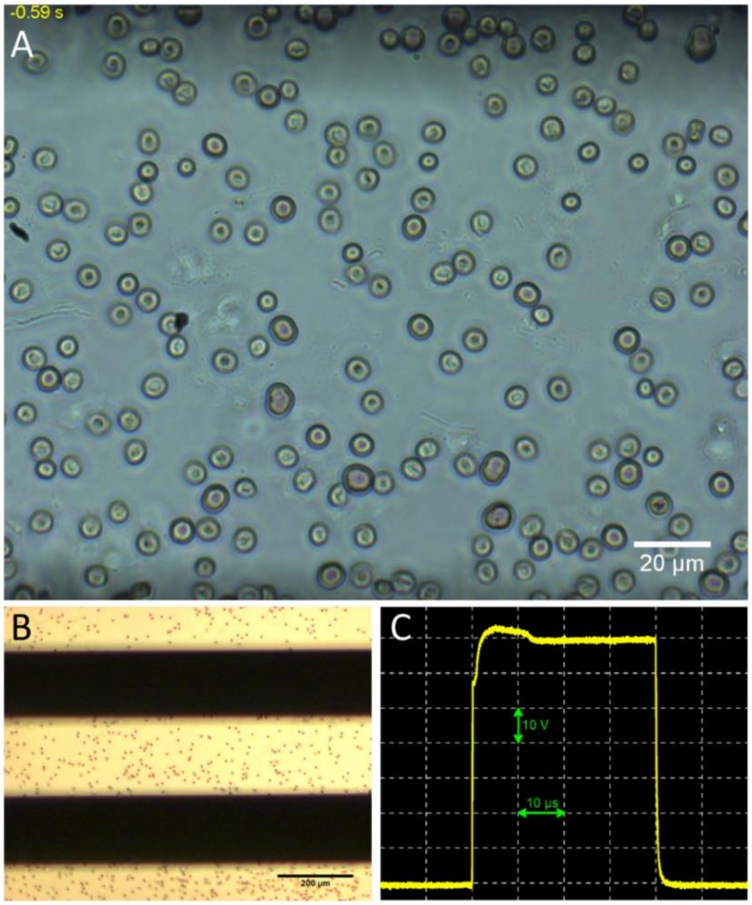


**Figure S1.** Overview of the electroporation procedure. A) Phase-contrast image of human RBCs suspended in isotonic sorbitol solution placed in an electroporation chamber. Prior to electropulsing, most RBCs display biconcave, discoid shape. The concave indentations or dimples in the membrane of isotonic RBCs appear in phase-contrast images as a dark central area surrounded by a bright ring corresponding to the thick perimetric region of a discoid RBC (see Figure 1A). B) The chamber consists of two parallel cylindrical wire electrodes with a diameter 200 µm. The electrodes are spaced by 200 µm. C) Typical square-wave electric pulse (~70 V, 40 µs) applied to the cell suspension.

The applied pulse voltage ($U_{app}$, Supplemental Fig. S1C) was measured by means of a 500-MHz digital storage oscilloscope (Tektronix, TDS 3052, Wilsonville, OR). From these signals, the field strength $E_0$ applied to the cell suspension was calculated as $U_{app}$ divided by the distance between the electrodes (200 µm). Given that the effective radius of human RBCs is $a_0$ = 3.34 µm (Lim et al., 2008), the applied field strength of $E_0$ = 3.5 kV/cm is nearly twice as high as the critical field strength required to induce the breakdown voltage of 1 V across the plasma membrane areas facing the electrodes, $U = 1.5 \times a_0 \times E_0$ = 1.75 V (Zimmermann et al., 2000).

## Image-based velocimetry analysis of hemolyzing RBCs

Tracking of hemolyzing RBCs was performed using the image-processing software ImageJ (NIH, Bethesda, MD). Briefly, in each video sequence, several regions of interest (ROIs) containing single RBCs were randomly selected and extracted as separate image stacks (Fig. S2A). Automated cell tracking, yielding the X,Y-coordinates of the center of mass and the projected cell area ($A_{proj}$), was implemented with an in-house developed ImageJ macro based on a simple thresholding method for cell edge detection (Fig. S2BC).

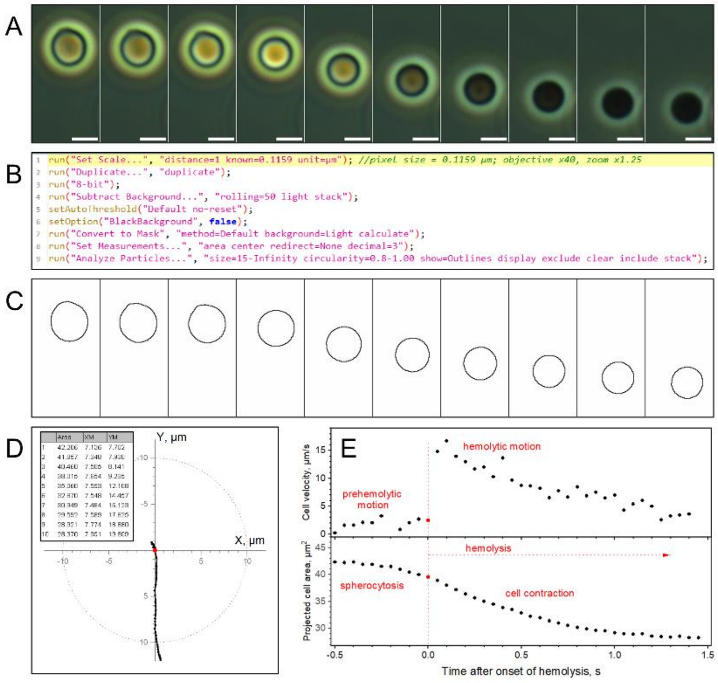


**Figure S2.** A representative example of the image-based tracking velocimetry of a hemolyzing RBC. A) Phase-contrast micrographs extracted from a video of a cell displaying hemolytic movement. Scale bar 5 µm. B) The ImageJ macro script used to detect the cell area and the coordinates of the cell center of mass. C) Cell edge detected by the macro applied to the image stack. D) Cell trajectory generated with the data of the center of mass (inset), gained with the macro. E) Time-dependent cell velocity and the corresponding changes in the projected cell area during hemolysis. Red data points in *D* and *E* denote the onset of hemolysis.

Trajectories of individual cells were plotted by setting the position of each cell at the onset of hemolysis to the origin of a Cartesian coordinate system (Fig. S2D). Only cells displaying linear, i.e., unidirectional, hemolytic motion were further evaluated. As evident from the cell trajectory (Fig. S2D), linearity persisted almost over the entire hemolytic cell motion, including the acceleration and high-velocity phases. Slight deviations from linearity occur only at the very late, i.e., low-velocity, stage of hemolytic motion, apparently due to some departure from sphericity and the associated non-radial jet ejection. From the acquired linear trajectories, cell displacements between two successive frames were calculated as $\Delta D_i = Sqrt((x_i - x_{i-1})^2 + (y_i - y_{i-1})^2)$ , where $x_i$ and $y_i$ are the x- and y-positions of the cell in frame *i*. The cell velocity $v_{cell}(t)$ in µm/s (Fig. S2E) was calculated as $v_{cell}(t) = \Delta D_i(t)/\Delta t$, where $\Delta t$ is the time interval between two frames. Usually, tracking of 40-50 hemolyzing RBCs was performed in at least five independent experiments. To allow for the cell-to-cell variability in the time-lag between electroporation and the onset of hemolysis, the time courses of cell velocity and displacement were averaged by synchronizing the time points of maximum cell acceleration.

## Computation, data analysis and statistics

The cell velocity and displacement data of hemolyzing RBCs are presented as mean (± SD) as function of time. Graph plotting and statistical analyses were performed using Origin 8.5 (Microcal, Northampton, MA). Statistical comparisons were made using Student’s t test for unpaired samples, with a level of significance set at $P < 0.01$. The ruling equations of the self-propelled RBC motion, which constitute the elastic-shell and fiber models and the general osmo-elastic model developed here, were solved numerically using the built-in *ParametricNDSolveValue* function of Wolfram Mathematica 12. The ruling equations were fitted to the experimental velocity data using the *NonlinearModelFit* Mathematica function.

## Ghost radius

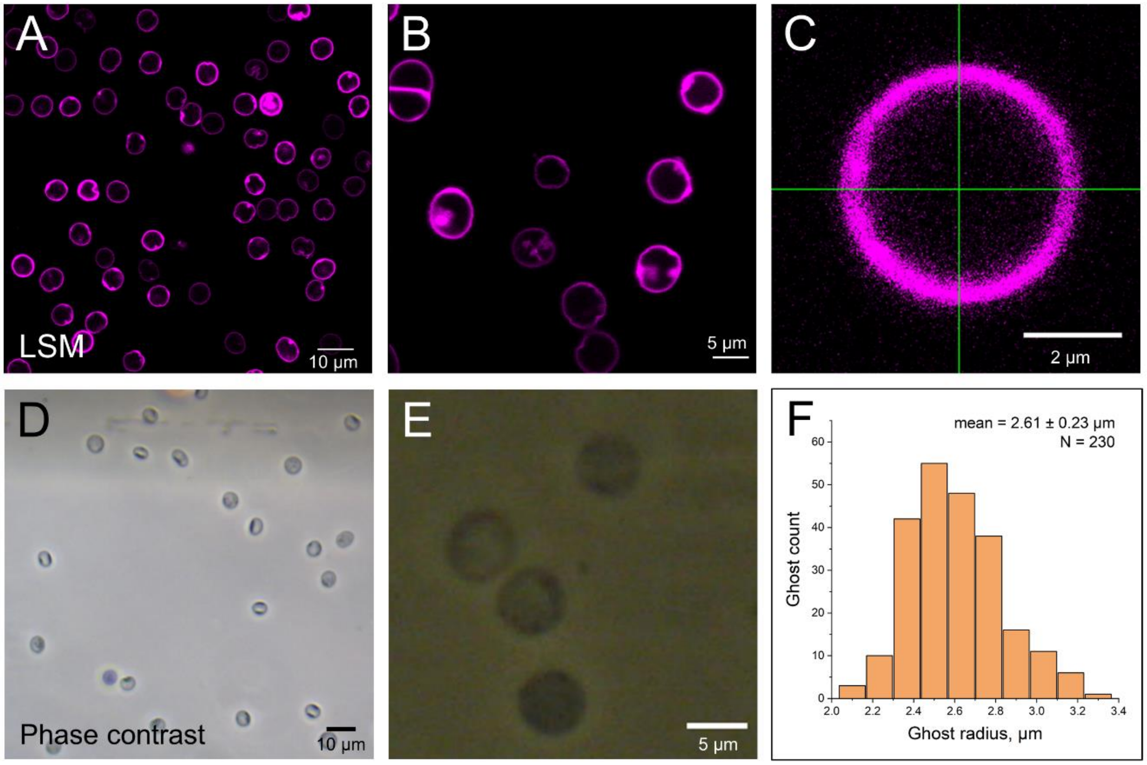


**Figure S3.** Microscopic measurement of ghost radii. Ghosts generated via electroporation were visualized by laser-scanning (A-C) and phase-contrast microscopy (D and E). F) The two imaging techniques yielded a mean (±SD) ghost radius of 2.61±0.23 μm (N = 230), corresponding to a ghost volume of ~75 fL.

## Statistical comparison of the velocity data

To compare the peak velocities ($v_{max}$) of ATP- and blebbistatin-treated cells to the respective controls (Fig. 4A and 4D), we applied the Student’s t-test for statistical significance. Cells treated with 1% DMSO (= dimethyl sulfoxide) served as control to blebbistatin-treated cells. The results of statistical comparison are shown in Fig. S4.

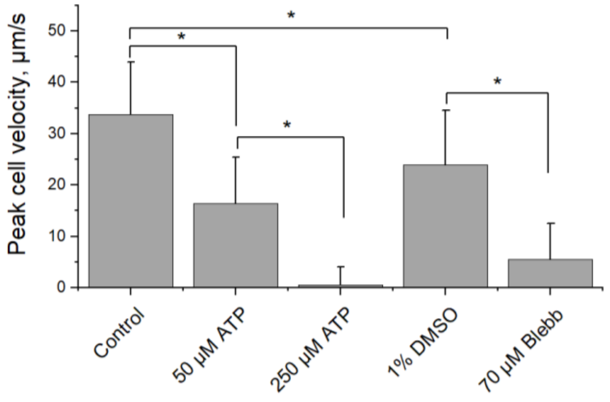


**Figure S4.** The bar graph summarizes the impact of drug treatment on the peak velocity of hemolyzing cells expressed in µm/s. Each bar represents the mean peak velocity ± SD of at least 40 cells measured in three independent experiments. The results were compared by Student's t-test for unpaired samples: * means $P < 0.01$.

As evident from Fig. S4 the differences in the peak velocity values between all tested conditions were statistically significant ($P < 0.01$).

## 2. THEORY AND ANALYTICAL RESULTS

### Limitations of the minimal elastic model

To gain an initial insight into the molecular mechanisms of the Hb-jet emission we developed a minimal elastic model consisting of Eqs. 1-6 given in the main text. The minimal model applies only to the self-propelled hemolytic cell motion, which is caused by the elastic contraction of the RBC membrane leading to ejection of the pressurized Hb-rich cytosol. Being valid only during hemolysis, the minimal model does not account for the prehemolytic cell volume changes, which are driven by the diffusive/osmotic fluxes of solutes and water through the electroporated membrane. Accordingly, the minimal model cannot explain the mechanism of intracellular pressure generation responsible for the Hb-jet expulsion. The generation of cytosolic pressure is addressed in a more general osmo-elastic model given in this Supplement below.

### Derivation of the ruling equation of motion (Eq. 1)

In accordance with Newton's laws of motion, a cytosolic jet from a hemolyzing RBC (Fig. 3) produces thrust on the cell by expelling the intracellular mass, *i.e.*, the Hb-rich cytosol serving as the propellant. The general equation of momentum conservation for a spherical cell exhibiting self-propelled motion can be written as follows:

$$\underbrace{\frac{d}{dt}(m_{cell}v_{cell})}_{\text{momentum changing rate of RBC}} + \underbrace{\overbrace{(v_{cell} - v_{jet})}^{\text{absolute jet velocity}} \times \overbrace{\rho v_{jet} S_0}^{\text{exhaust mass flow}}}_{\text{momentum changing rate of cytosol outflow}} = - \underbrace{6\pi a \eta v_{cell}}_{\text{external viscous drag force}} \quad \text{(S1).}$$

The first and second terms on the LHS of Eq. S1 expand, respectively, to

$$\frac{d}{dt}(m_{cell}v_{cell}) = m_{cell}\frac{dv_{cell}}{dt} + v_{cell}\overbrace{\frac{dm_{cell}}{dt}}^{\text{mass changing rate}} \quad \text{(S2),}$$

and

$$(v_{cell} - v_{jet}) \times \rho v_{jet} S_0 = v_{cell}\overbrace{\rho v_{jet} S_0}^{\text{exhaust mass flow}} - v_{jet}\rho v_{jet} S_0 \quad \text{(S3).}$$

The mass changing rate of RBC on the RHS of Eq. S2 is clearly equal to the mass efflux/exhaust

flow by the hemolytic jet in Eq. S3, where $v_{jet}$ the jet velocity relative to the cell. Consequently,

$$dm_{cell}/dt = -\rho v_{jet} S_0, \quad \text{(S4)},$$

where $v_{jet}$ the jet velocity relative to the cell and $dm_{cell}/dt < 0$, i.e., the cell mass decreases with time. Substitution of Eq. S4, simplifies Eq. S1 to Eq.1 given in the main text:

$$\underbrace{m_{cell}\frac{d}{dt}(v_{cell})}_{\substack{\text{inertial term}\\ \text{of the cell}}} = \underbrace{\rho v_{jet}^2 S_0}_{\substack{\text{thrust force}\\ \text{by the jet}}} - \underbrace{6\pi a\eta v_{cell}}_{\substack{\text{viscous drag}\\ \text{force}}} \quad \text{(1)},$$

or an equivalent equation for a spherical cell of radius $a$:

$$\underbrace{\rho\frac{4}{3}\pi a^3}_{\text{cell mass}}\frac{d}{dt}(v_{cell}) = \rho v_{jet}^2 S_0 - 6\pi a\eta v_{cell} \quad \text{(1a)}.$$

In addition to the ruling equation of motion (Eq. 1), the minimal elastic model also includes Eqs. 2-5 (or 6) given in the main text. According to Eq. 2, cell volume decreases only due to the convective efflux of the cytosol through the hemolytic hole, while diffusive/osmotic fluxes through the membrane are neglected. The acceleration of hemolyzing RBCs (Fig. 4A) at the onset of Hb-jet ejection can only result from a rapid expansion of a small prehemolytic hole in the bilayer (at the cell pole facing the cathode) to a large hemolytic hole permeable for Hb-molecules. Membrane pore/hole expansion modelled by an exponential function (Eq. 3). Given that the Hb radius $r_{\mathrm{Hb}}$ = 3.1 nm, $S_{\mathrm{hole}}^{\mathrm{max}} = \pi \times r_{Hb}^2 \approx 30\ \mathrm{nm}^2$. The time constant of the hole expansion was determined by fitting the elastic model to the RBC velocity data (Fig. 5C).

## Justification of the use of Bernoulli's equation

The Bernoulli equation for an inviscid-like flow (Eq. 4) relates the Hb-jet exit velocity to the cytosolic pressure $\Delta p_{\mathrm{cyt}}$. In addition to the Bernoulli equation, we also examined the Roscoe equation (Roscoe, 1949): $v_{jet} = \Delta p_{\mathrm{cyt}} r_{\mathrm{pore}}/3\pi\eta_{\mathrm{cyt}}$, where $\eta_{\mathrm{cyt}}$ = ~2 mPa s is the viscosity of the cytosol for our experimental conditions. The Roscoe equation infers a pressure drop in the viscous flow of the cytosol through the hemolytic hole modelled as a circular orifice in an infinitely thin wall. As shown in Fig. S5, substitution of the Roscoe equation instead of the Bernoulli equation into our elastic model reduces by 3-4 orders of magnitude the cell velocity. The near-vanishing of the propulsive force on the cell suggests a quasi-inviscid flow of the Hb-jet through the hemolytic hole.

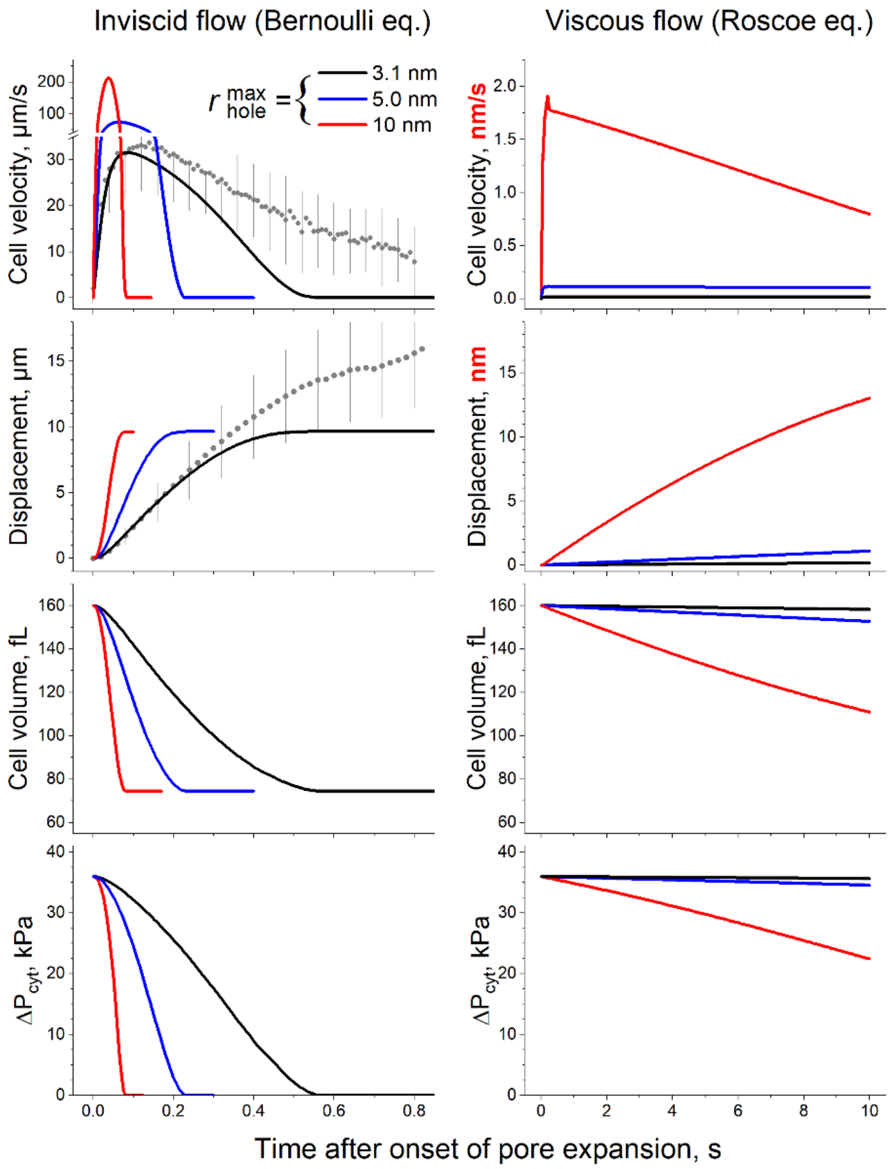


**Figure S5.** Numerical simulations of the elastic-fiber model suggest an inviscid-like cytosol outflow through the hemolytic hole. Curves in the left column were calculated assuming inviscid cytosol flow according to Bernoulli equation (Eq. 4), whereas the right column shows curves calculated assuming viscous cytosol flow (Roscoe equation: $v_{jet} = \Delta p_{\mathrm{cyt}} r_{\mathrm{pore}} / 3\pi\eta_{\mathrm{cyt}}$ (Roscoe. 1949). Black, blue and red curves were calculated for the hemolytic hole radius of 3.1, 5 and 10 nm, respectively. The best fit to the experimental cell velocity and displacement data (symbols, upper left graphs) were obtained for the hole radius 3.1 nm and inviscid cytosol flow. For other relevant parameters refer to Fig. 5C. In contrast, a viscous flow would completely abolish any detectable hemolytic cell motion by reducing the cell velocity from the µm/s to the nm/s range (upper right graphs).

## Derivation of the elastic constitutive equations (Eqs. 5 and 6)

The driving force for the Hb-jet ejection is provided by the cytosolic overpressure $\Delta p_{cyt}$ generated by elastic expansion in the composite RBC membrane. To account for both components of the membrane (i.e., the bilayer and the cytoskeleton), we derived two different elastic constitutive equations. Based on the area elasticity theory (Helfrich, 1973), Eq. 5 treats the membrane bilayer as a 2D elastic shell and relates $\Delta p_{cyt}$ to the area elasticity modulus of the shell $K_{shell}$ and the cell radius $a$. Equation 6 considers a cytoskeletal meshwork consisting of $N_f$ elastic fibers firmly anchored *at both ends* to the bilayer.

Equation 5 in the elastic-*shell* model relates the cytosolic overpressure $\Delta p_{cyt}$ to the tension in the membrane bilayer. For the derivation of Eq. 5, the RBC membrane bilayer is viewed as an elastic spherical shell with a variable radius $a$ and an areal elasticity modulus $K_{shell}$. The shell is assumed to undergo isotropic spherical expansion from its relaxed state (radius $a_0$) to a swollen tensed state (radius $a$), as illustrated in Fig. S6.

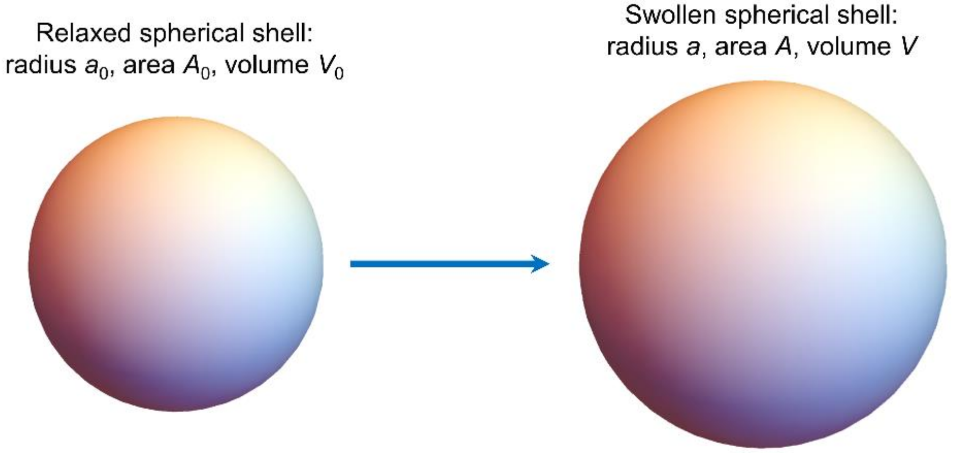


**Figure S6.** Isotropic expansion of an elastic spherical shell from its initial relaxed state to a swollen tensed state. The initial shell radius a0 denotes the radius of a fully relaxed/contracted shell with zero tension (subscript "0"). The initial surface area and volume are $A_0 = 4\pi a_0^2$ and $V_0 = 4\pi a_0^3/3$, respectively. Symbol a stands for the radius of a tensed membrane shell.

According to Helfrich (1973), the elastic energy of a shell per unit area $w$ is:

$$w = \frac{K_{shell}}{2}\left(\frac{A - A_0}{A_0}\right)^2 \qquad \text{(S5)},$$

where $K_{\text{shell}}$ is the area elasticity modulus [N/m], $A$ is the surface area of a tensed membrane shell and $A_0$ is the surface area, at which the elastic stress by area expansion is zero.

Accordingly, the total elastic energy $E$ [J] of a spherical shell with isotropic $K_{\text{shell}}$ can be expressed as:

$$E = 4\pi a^2 w = 4\pi a^2 \frac{K_{\text{shell}}}{2}\left(\frac{4\pi a^2 - 4\pi a_0^2}{4\pi a_0^2}\right)^2 = 4\pi a^2 \frac{K_{\text{shell}}}{2}\left(\frac{a^2 - a_0^2}{a_0^2}\right)^2 \qquad \text{(S6)},$$

where $4\pi a^2 \equiv A$ and $4\pi a_0^2 \equiv A_0$ are defined above.

If the shell area expansion is caused only by the pressure difference between intra- and extracellular space, $\Delta p_{\text{cyt}}$, the amount of mechanical work $W$ required to expand the shell from the radius $a_0$ to $a$ is:

$$W = \int_{4\pi a_0^3/3}^{4\pi a^3/3} \Delta p_{cyt}\, dV \qquad \text{(S7)}$$

Neglecting the power loss during shell expansion, the work $W$ should be equal to the total elastic energy $E$ ($W$=$E$) for an arbitrary radius $a$. Thus, the relationship between the intracellular overpressure $\Delta p_{\text{cyt}}$ [Pa] and $E$ can be derived by differentiating $E$ by $V$:

$$\Delta p_{\text{cyt}} = \frac{\partial W}{\partial V} = \frac{\partial E}{\partial V} = \frac{\partial E}{\partial a}\frac{\partial a}{\partial V} = \frac{\partial E}{\partial a}\Big/\frac{\partial V}{\partial a} = \frac{2\pi K_{\text{shell}}}{4\pi a^2}\left\{\left(\frac{a}{a_0}\right)^2 - 1\right\}\left[2a\left\{\left(\frac{a}{a_0}\right)^2 - 1\right\} + \frac{4a^3}{a_0^2}\right] \qquad \text{Eq. S8.}$$

Equation S8 simplifies to:

$$\Delta p_{\text{cyt}} = \frac{K_{\text{shell}}}{a}\left\{\left(\frac{a}{a_0}\right)^2 - 1\right\}\left\{3\left(\frac{a}{a_0}\right)^2 - 1\right\} \qquad \text{Eq. S9,}$$

which represents the elastic constitutive equation (Eq. 5) used in the elastic-shell model.

To derive the elastic constitutive equation used in the elastic-*fiber* model (Eq. 6), we assume that the RBC elasticity is dominated by the cortical cytoskeleton consisting of $N_{\text{fiber}}$ elastic fibers anchored at both ends to the bilayer. In case of the actin-spectrin cytoskeleton, the interconnected spectrin

filaments form a regular continuous meshwork (Fig. S7A), whereas separate NM2 filaments are randomly distributed over the inner surface of the RBC membrane (*see* Fig. 5BC, main text).

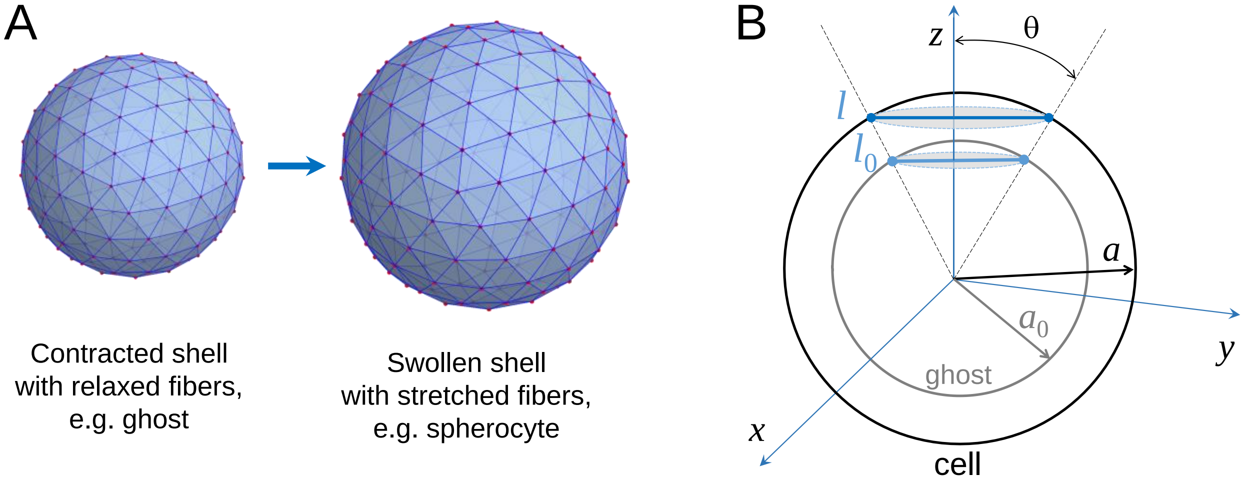


**Figure S7.** Isotropic spherical expansion of a cortical cytoskeleton. **A**: The triangular network illustrates an arbitrary fiber meshwork, such as the actin-spectrin cytoskeleton. **B**: The spherical chords (blue lines) symbolize an elastic fiber firmly anchored at both ends to the bilayer membrane. Symbols $l_0$ and l denote, respectively, the relaxed and stretched states of the fiber, corresponding, to a contracted ghost (radius $a_0$) and a swollen RBC spherocyte (radius a).

Each fiber is firmly connected at both ends, in a chord-like manner (blue lines in Fig. S7B), to the cytosolic bilayer surface. The stiffness or spring constant $k_f$ of a single fiber is defined as a proportionality constant between the applied force $F$ and strain $\Delta l = l - l_0$:

$$F = k_f \cdot \Delta l = k_f \cdot (l - l_0) \quad \text{(S10)},$$

Thus, the elastic energy $w$ of a single stretched filament can be expressed as:

$$w = \int_{l_0}^{l} k_f (l - l_0) dl = \frac{k_f}{2}(l - l_0)^2 \quad \text{(S11)},$$

where $l$ stands for the length of a stretched fiber in a swollen RBC of radius $a$ at the onset of hemolysis and $l_0$ denotes the fiber length in a fully relaxed stress-free state, i.e., in a ghost of radius $a_0$ at the end of hemolytic motion. Accordingly, the total elastic energy $E$ of $N_f$ fibers in a swollen RBC can be expressed as:

$$E = N_f \frac{k_f l_0^2}{2} \left(\frac{l - l_0}{l_0}\right)^2 \quad \text{(S12)}.$$

If cell swelling from the relaxed ghost state of volume $V_0 = 4\pi a_0^3/3$ to a volume $V = 4\pi a^3/3$ is driven

only by the cytosolic overpressure, $\Delta p_{\text{cyt}}$, the following relation holds between $E$, $\Delta p_{\text{cyt}}$ and $V$:

$$E = \int_{V_0}^{V} \Delta p_{\text{cyt}} dV = N \frac{k_f l_0^2}{2} \left(\frac{l-l_0}{l_0}\right)^2 \qquad \text{(S13).}$$

Taking into account that the sphere chord length $l$ scales linearly with the shell radius $a$, i.e. $(l - l_0)/l_0 = (a-a_0)/a_0$, differentiation of $E$ in Eq. S13 by cell volume $V$ gives the following expression for $\Delta p_{\text{cyt}}$:

$$\Delta p_{\text{cyt}} = \frac{dE}{dV} = N_f k_f l_0 (\frac{l-l_0}{l_0}) \frac{dl}{da} \frac{da}{dV} = N_f k_f l_0 (\frac{a-a_0}{a_0}) \frac{dl}{da} \frac{da}{dV} \qquad \text{(S14).}$$

Given that $l/l_0 = a/a_0$ and $V$=$4\pi a^3/3$, and therefore $\mathrm{d}l/\mathrm{d}a = l_0/a_0$ and $\mathrm{d}a/\mathrm{d}V = (4\pi a^2)^{-1}$, the RHS of Eq. S14 transforms to Eq. S15 (or Eq. 6 in the main text) used in the elastic-fiber model:

$$\Delta p_{\text{cyt}} = \frac{N_{\text{f}}}{4\pi} k_{\text{f}} \left(\frac{l_0}{a_0}\right)^2 \frac{a_0}{a^2} (\frac{a}{a_0} - 1) \qquad \text{(S15).}$$

From Eq. S15, the total elasticity of a fiber network can be defined as:

$$K_{\text{tf}} = \frac{N_f \cdot k_f}{4\pi} \left(\frac{l_0}{a_0}\right)^2 \qquad \text{(S16),}$$

where the the total network elasticity $K_{\text{tf}}$ with the dimension of [J/m$^2$] or [N/m] is analogous to the area elasticity modulus $K_{\text{shell}}$ in Eqs. 5 and S9 in the elastic shell model.

## General osmo-elastic model (gOEM)

Using our minimal elastic-fiber model we provide strong evidence for the involvement of NM2 filaments in the propulsive behavior of hemolyzing RBCs (Fig. 6C). However, the minimal elastic model considers only the contraction of stretched NM2 filaments during hemolytic motion without explaining the mechanism of the filament stretching during the prehemolytic phase leading to the cytosolic pressure generation. We therefore developed a more general osmo-elastic model (gOEM), which accounts not only for the hemolytic cell motion, but also addresses the mechanism of the osmotic volume changes of electroporated RBCs, including both the transient shrinkage and the following swelling to hemolytic volume observed in experiments (Fig. 1).

Based on the reported data for the RBC solutes (Gary-Bobo and Solomon, 1968), our gOEM considers two major groups of intracellular osmolytes differing in their molecular size and therefore in their permeability through electropores. The first and the most abundant group of solutes with an overall osmolality $C_{el}$ of ~250 mOsm includes small ionic species $K^+$, $Cl^-$, $HPO_4^{-2}$/$H_2PO_4^{-1}$, etc., hereafter called “electrolytes”. Due to their small Stoke’s radii $r_S$ of 0.16-0.32 nm, these inorganic ions, can pass freely through electropores, whose radii vary between ~0.2 and 0.8 nm (Saulis and Saule, 2012). Electropores are also permeable for ATP ($r_{ATP} \approx 0.7$ nm). The second group of cytosolic solutes includes large *colloidal* molecules including hemoglobin (Hb) and other cytosolic proteins. Because of its large radius of ~3.1 nm, a Hb molecule cannot pass through the initial electropores and can be ejected only through the hemolytic hole during hemolysis. In our experiments, the extracellular solution consisted mainly of ~300 mOsm sorbitol, whose Stoke’s radius of ~0.4 nm.

Extending the previously reported osmotic model (Shirakashi et al., 2004) by the convective leak-out of the pressurized cytosol through the pre- and hemolytic holes, the electroporation-mediated cell volume changes can be modelled by the ruling equations S17-S22. These equations are based on the classical two-parameter $L_p$-$P$-model (Kleinhans, 1998) and express the mass conservation of the relevant solutes and water:

$$\overbrace{d\left(C_{sor}^{i}V\right)/dt}^{J_{\mathrm{sor}}} = \overbrace{P_{sor}A_0\left(C_{sor}^{o} - C_{sor}^{i}\right)}^{\text{diffusion through electropores}} - \overbrace{C_{sor}^{i}v_{jet}S_{hole}}^{\text{convective leak-out}} \quad \text{(S17)},$$

$$\overbrace{d\left(C_{el}^{i}V\right)/dt}^{J_{\mathrm{el}}} = P_{el}A_0\left(C_{el}^{o} - C_{el}^{i}\right) - C_{el}^{i}v_{jet}S_{hole} \quad \text{(S18)},$$

$$\overbrace{d\left(C^i_{\mathrm{ATP}}V\right)/dt}^{J_{\mathrm{ATP}}} = P_{el}A_0\left(C^o_{\mathrm{ATP}} - C^i_{\mathrm{ATP}}\right) - C^i_{\mathrm{ATP}}v_{jet}S_{hole} \qquad \text{(S19).}$$

The ruling Eqs. S17-S19 define the transmembrane fluxes $J$ [mol/s] of sorbitol, electrolytes and ATP denoted by subscripts "*sor*", "*el*", and "ATP", respectively. Symbol $C$ stands for the osmotic concentrations [mOsm]. Superscripts "$i$" and "$o$" denote intra- and extracellular concentrations, respectively. The cell volume $V = 4\pi a^3/3$, where $a$ is the time-varying cell radius. The permeability coefficients $P$ [m/s], the hydraulic conductivity $L_p$ [$m^3$/(N·s)] and the membrane area $A_0$ = 140 μ$m^2$ are assumed to be time invariable. The fluxes of the three small solutes consist of the diffusion through electropores and the convective leak-out (or jet) through the (pre-) hemolytic hole, whose area $S_{\mathrm{hole}}$ increases abruptly at the onset of hemolysis (Eq. 3). Although the contribution of ATP to intracellular osmolality is negligible (<1 mOsm), Eq. S19 is necessary for modelling the ATP depletion in electroporated RBCs.

In contrast to the small solutes, the efflux of colloidal solutes occurs only during hemolysis via their convective leak-out through the hemolytic hole:

$$\overbrace{d\left(C^i_{col}V\right)/dt}^{J_{\mathrm{col}}} = -\overbrace{C^i_{col}v_{jet}S_{hole}}^{\substack{\text{convective}\\ \text{leak-out}}} \qquad \text{(S20).}$$

Changes of the RBC volume ($J_V$) occur via the osmotic water flux ($J_{\mathrm{osm}}$) through the membrane and via the convective cytosol leak-out ($J_{\mathrm{jet}}$) through the pre- and hemolytic holes:

$$J_V = \overbrace{dV/dt}^{\substack{\text{cell volume}\\ \text{change}}} = J_{osm} - \overbrace{v_{jet}S_{hole}}^{\substack{\text{cytosol jet}\\ J_{\mathrm{jet}}}} \qquad \text{(S21).}$$

The osmotic water flux $J_{\mathrm{osm}}$ is governed by Eq. S22, which allows for both the osmotic ($\Delta\pi_{\mathrm{cyt}}$) and the hydrostatic ($\Delta p_{\mathrm{cyt}}$) pressure differences across the membrane:

$$J_{osm} = L_pA_0\left\{\overbrace{RT\left(C^i_{sor} + C^i_{el} + C^i_{\mathrm{ATP}} + C^i_{col} - C^o_{sor} - C^o_{el} - C^o_{\mathrm{ATP}} - C^o_{col}\right)}^{\text{osmotic pressure, }\Delta\pi_{\mathrm{cyt}}} - \Delta\boldsymbol{p}_{cyt}\right\} \qquad \text{(S22).}$$

Before the onset of hemolysis, the osmotic water flux ($J_{\mathrm{osm}}$) dominates over the pressure-driven leak-out of the cytosol ($J_{\mathrm{jet}}$) through the prehemolytic hole ($J_{osm} \gg J_{jet}$), whereas during hemolysis after the hole expansion $J_{jet} \gg J_{osm}$. Equation S22 also implies that the hydrostatic pressure in the cytosol $\Delta p_{\mathrm{cyt}}$ opposes the osmotically driven water influx.

Applying the product rule to the LHS of Eqs. S17-S19 and substituting Eq. S21 (i.e., $dV/dt = J_{osm} - v_{jet}S_{hole}$) into Eqs. S17-S19 yields the following simplified expressions for the small solute concentrations:

$$V\, dC_{sor}^{i}/dt = -C_{sor}^{i} \times J_{Osm} + P_{sor}A_0(C_{sor}^{o} - C_{sor}^{i}) \quad \text{(S23)},$$

$$\mathrm{V}\, dC_{el}^{i}/dt = -C_{el}^{i} \times J_{Osm} + P_{el}A_0(C_{el}^{o} - C_{el}^{i}) \quad \text{(S24)},$$

$$V\, dC_{\mathrm{ATP}}^{i}/dt = -C_{\mathrm{ATP}}^{i} \times J_{Osm} + P_{\mathrm{ATP}}A_0(C_{\mathrm{ATP}}^{o} - C_{\mathrm{ATP}}^{i}) \quad \text{(S25)}.$$

As mentioned above (Eq. S20), the total amount of the colloidal solutes ($C_{col}^{i}V$) in the cytosol remains unchanged during the prehemolytic phase (Eq. S20). Therefore, the following equation holds:

$$d\left(C_{col}^{i}V\right)/dt = 0. \quad \text{(S26)},$$

which implies that before the onset of hemolysis the intracellular colloid *concentration* varies with time only due to the osmotic cell volume changes $C_{col}^{i}(\mathrm{t}) = C_{col}^{i}(0) \times V_{iso}/V$ , where $C_{col}^{i}(0)$ is the initial colloid concentration in isotonic RBCs of volume $V_{\mathrm{iso}}$ =100 fL.

During hemolysis, the ruling equation S20 for colloidal solutes transforms to:

$$VdC_{col}^{i}/dt = -\,C_{col}^{i}(J_{osm} - v_{jet}S_{\mathrm{hole}}) \quad \text{(S27)},$$

where the term $C_{col}^{i}J_{osm}$ on the RHS stands for the osmotic water flux during hemolysis.

Finally, to allow for the effect of the osmotic water flux on the cell mass, an additional innertial term $\rho v_{cell} \times J_{Osm}$ was intoduced into the force balance equation (Eq. 1, main text):

$$\underbrace{m_{cell}\frac{d}{dt}(v_{cell}) + \rho v_{cell} \times J_{osm}}_{\text{inertial term of the cell}} = \rho v_{jet}^{2}S_{hole} - 6\pi a\eta v_{cell} \quad \text{(S28)}.$$

Taken together, the gOEM comprises the above equations S22-S28, along with the pore expansion equation (Eq. 3), the Bernoulli equation (Eq. 4) and the elastic constitutive equation for elastic fibers (Eq. 6) given in the main text. The system of differential equations constituting the gOEM was solved numerically for the cell velocity, volume, $\Delta p_{\mathrm{cyt}}$ and other relevant quantities, using the built-in *ParametricNDSolveValue* function of Wolfram Mathematica. The gOEM equations were solved by applying the following initial conditions (*time* = 0) for the intracellular solute concentrations: $C_{col}^{i} = 50$ mOsm , $C_{el}^{i} = 250$ mOsm , $C_{\mathrm{ATP}}^{i} = 1$ mOsm , and $C_{sor}^{i} = 0$ mOsm . The extracellular sorbitol concentration was $C_{sor}^{o} = 300$ mOsm. Because of the very small cell volume percentage in the RBC samples, the efflux-associated changes of the electrolyte, ATP and colloid concentrations in the

suspending medium were neglected, i.e., $C_{el}^{o} = C_{\mathrm{ATP}}^{o} = C_{col}^{o} = 0$ mOsm. The RBC membrane area and volumes were taken from the literature: $A_0$ = 140 μm$^2$, $V_{\mathrm{disc}}$ = 100 fL, $V_{\mathrm{ech}}$ =75 fL, and $V_{\mathrm{crit}}$ = 160 fL (Lim et al., 2008; Chen, 2002). The ghost volume $V_{\mathrm{ghost}}$ = 75 fL (*see* Fig. S2). The parameters of NM2 filaments and hemolytic hole were taken from the best-fit of the minimal elastic model to the hemolytic motion data (Fig. 5C). The unknown osmotic parameters, i.e., $L_{\mathrm{p}}$, $P_{\mathrm{el}}$, $P_{\mathrm{sor}}$, and $P_{\mathrm{ATP}}$, were found by fitting the gOEM to the prehemolytic RBC volume changes (see below).

## Comparison between experiments and the general osmo-elastic model

The gOEM describes fairly well the entire electrohemolysis, including both the osmotically driven prehemolytic cell volume changes (Fig. S8A) and the hemolytic cell motion (Fig. S9). The volume curve in Fig. S8A (solid blue-red) was generated by adjusting the osmotic parameters to match the volume data (circles) during RBC transformation from a discocyte with the initial volume of 100 fL to an echinocyte (~75 fL), and finally to a spherocyte with a critical hemolytic volume of ~160 fL. The hemolytic volume is achieved 8 s after electroporation, which corresponds to the experiment illustrated in Fig. 1.

As seen in Fig. 1, an electroporated RBC first undergo transient shrinkage and echinocytosis. The observed cell shrinkage (Fig. 1D) indicates that shortly after electroporation the efflux rate of small cytosolic electrolytes (radius ≈ 0.16-0.32 nm) exceeded the influx rate of sorbitol (~0.4 nm), which is consistent with the data reported elsewhere (Saulis and Saule, 2012; Sözer et al., 2017). A reasonable agreement between the experiment (Fig. 1) and the gOEM is achieved by assuming $P_{\mathrm{el}}$ = 14 μm/s, $P_{\mathrm{sor}}$ = 3.3 μm/s, $L_{\mathrm{p}}$ = 5×10$^{-12}$ m$^3$/(N·s). Changes of the intracellular solute concentrations responsible for the osmotic cell volume changes are illustrated in Fig. 6B. The electrolyte concentration rapidly decreases ($C_{el}^{i}$, blue line in Fig. S8B) due to their outward diffusion through electropores, whereas the inward diffusion of sorbitol leads to its accumulation in the cytosol ($C_{sor}^{i}$, red line). In contrast, the concentration of large impermeable colloidal solutes changes only slightly ($C_{col}^{i}$, green line).

According to the gOEM, the minimal cell volume is reached when the cytosol is fully depleted of electrolytes, i.e., ~0.25 s after electroporation (Fig. 6A and 6B). Thereafter, in agreement with experiments (Fig. 1), the gOEM predicts the onset of an enduring cell swelling (Fig. 6A), which is driven by the continuing influx of the abundant sorbitol along with the osmotic pressure imposed by cytosolic colloids (Eq. S22).

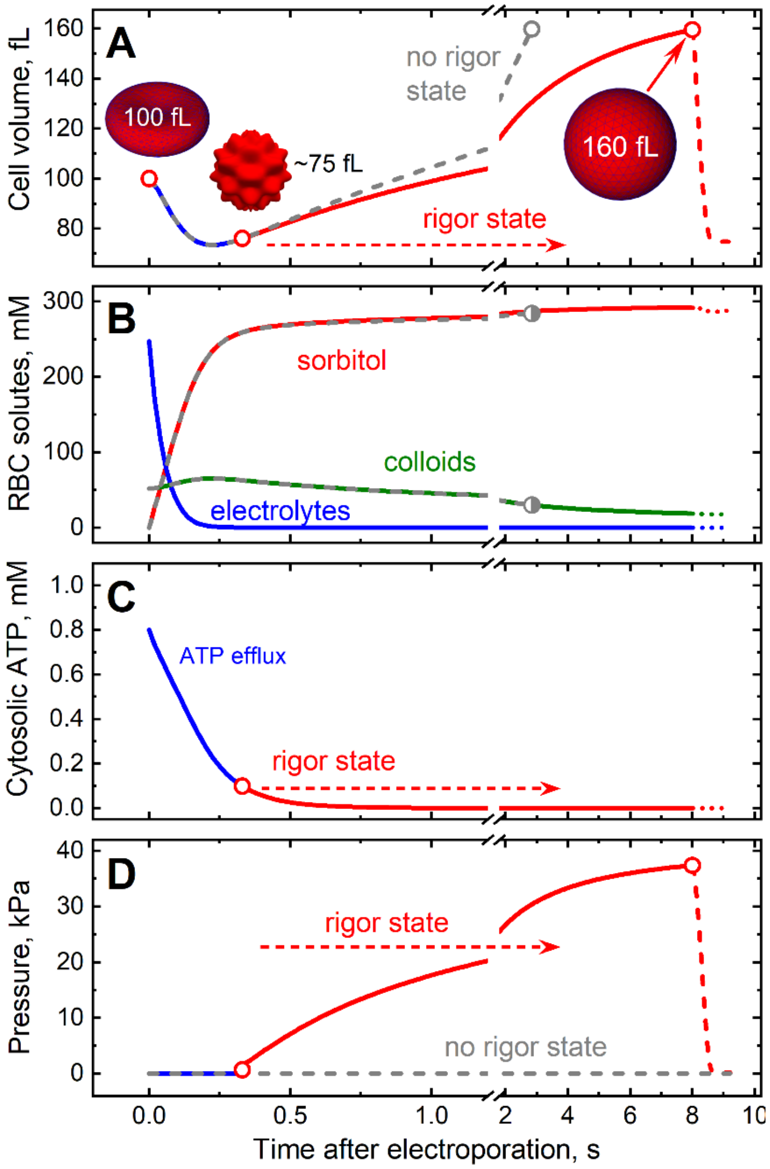


**Figure S8.** Theoretical curves calculated with the gOEM illustrate the prehemolytic changes (time < 8 s) in the cell volume (**A**), intracellular solute concentrations (**B**) and ATP (**C**), and cytosolic pressure (**D**), induced by electroporation. The osmotic parameters were adjusted to match the transient shrinkage/transformation of a discocyte (~100 fL) to an echinocyte (~75 fL), followed by swelling to the hemolytic volume (160 fL) achieved ~8 s after electroporation. The red-colored portions of the curves in **A, C** and **D** indicate rigid ATP-depleted RBCs ($ATP_{cyt}$ < 0.1 mM), whose membranes are stiffened by the rigor state actomyosin. Rigid cells are more resistant to osmotic swelling than flaccid RBCs (gray dashed line in **A**). Moreover, osmotic swelling generates a substantial hydrostatic pressure of up to ~35 kPa only in rigid cells (red line in **D**), but not in flaccid RBCs (gray line). This hydrostatic pressure in the cytosol provides the driving force for the Hb-jet ejection during hemolysis. Dashed portions of the curves at time > 8 s correspond to the hemolysis stage.

Although ATP contributes only slightly to the intracellular osmolality (<1 mOsm), the ATP depletion causes RBC stiffening and echinocytosis (Dreher et al., 1978; Clark et al. 1981). As proposed in the present study, echinocyte stiffening occurs due the formation of a rigid scaffold of NM2 filaments tightly locked in the rigor state to the actin nodes of the RBC membrane (Fig. 6). The ATP depletion can occur directly via its efflux through electropores. Moreover, the rapid efflux of small phosphate anions $HPO_4^{-2}/H_2PO_4^{-1}$ can also contribute to the ATP depletion by reducing its enzymatic regeneration from ADP and inorganic phosphate. In the gOEM simulations (Fig. S8), the NM2-actin rigor state is activated when the ATP concentration decreases to 0.1 mM and below ($time \geq 0.33$ s in Fig. S8), which corresponds to the ATP content in rigid echinocytes (Braunmüller et al. 2012). The gOEM curves in Fig. S8 were calculated by adjusting $P_{\mathrm{ATP}}$ to 4 µm/s.

The stiffening of echinocytes mediated the NM2-actin rigor state has great impact on the rate of cell swelling. Thus, a rigid cell requires much more time (~8 s) to reach hemolytic volume, as compared to a flaccid cell (~3 s) lacking rigor actomysion (Fig. S8A, gray dashed line). This theoretical result agrees well with the earlier findings that ATP-depleted echinocytes are very rigid cells with an increased resistance to osmotic swelling (Dreher et al., 1978; Clark et al. 1981). The resistance to osmotic swelling is apparently due to the generation of the hydrostatic pressure in the cytosol $\Delta p_{\mathrm{cyt}}$ (Fig. S8D), which opposes the osmotic water influx into electroporated RBCs (Eq. S22). Compared to a rigid RBC, a flaccid RBC not only swells much faster, but it also lacks cytosolic pressure (dashed lines in Fig. S8D). In contrast to the cell volume and cytosolic pressure, the kinetics of intracellular solute concentrations are practically not affected by the RBS membrane stiffening (gray dashed curves in Fig. S8B).

According to our NM2 rigor-state model, $\Delta p_{\mathrm{cyt}}$ is generated during osmotic swelling of echinocytes accompanied by flattening of spicules and stretching of NM2 filaments bound in a chord-like manner to the bilayer at the spicule base (Fig. 6B-6D). The stretching-induced tensile forces of NM2 filaments are balanced by the elastic bilayer tension ($\sigma_{\mathrm{BL}}$), which in turn generates intracellular pressure ($\Delta p_{\mathrm{cyt}}$, Fig. 6C) in accordance with the Laplace equation: $\Delta p_{\mathrm{cyt}} = \sigma_{\mathrm{BL}}/a_{\mathrm{cell}}$, where $a_{\mathrm{cell}}$ is the cell radius. The presence of a substantial $\Delta p_{\mathrm{cyt}}$ during cell swelling is evinced by the slow prehemolytic motion (Fig. 2B3). During this phase, the bilayer tension was apparently not sufficient to induce significant expansion of electropores. To match the observed prehemolytic velocity of ~2 µm/s, the prehemolytic hole radius of 0.6 nm was used for calculations of the gOEM curves in Fig. S8.

To sum up, the gOEM (Fig. S8) not only agrees well with the prehemolytic cell volume and shape

changes observed in experiment (Fig. 1A-1J), but it also predicts that osmotic swelling of rigid RBCs generates a substantial hydrostatic pressure of up to ~35 kPa in the cytosol (Fig. S8D). However, a bulk ejection of the pressurized cytosol can only occur when a sub-nm sized electropore expands to a sufficiently large hemolytic hole with a radius comparable to that of the Hb molecule (~3.1 nm).

An electropore expansion starts once an RBC reaches its critical hemolytic volume of ~160 fL. This is because further swelling generates a substantial mechanical tension in the membrane bilayer, which in turn initiates expansion of the largest electropore to a hemolytic hole, as illustrated in the inset of Fig. S9A. Figure S9 also compares the experimental cell velocity and volume data (symbols) with the theoretical curves calculated with the gOEM, using the same parameters as in Fig. S8.

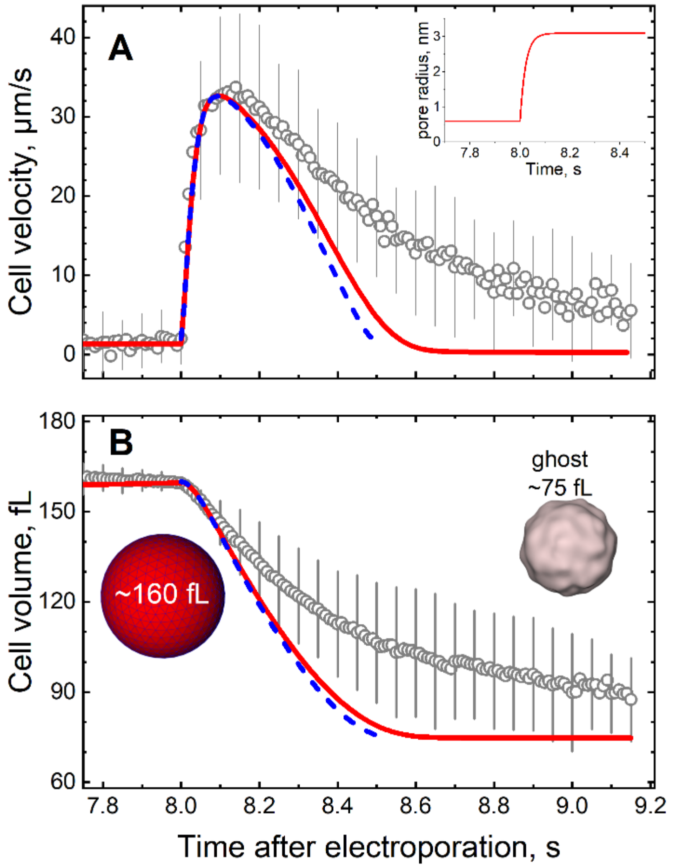


**Figure S9.** Fitting the general osmo-elastic model (gOEM) to the experimental cell velocity and volume data during hemolytic cell contraction and motion (**A** and **B**, red solid curves). Inset in **A** illustrates expansion of the hemolytic hole radius at the onset of hemolysis. For comparison, the best fit curves calculated with the minimal elastic model are also shown (blue dashed curves).

Taken together, the gOEM provides a good approximation to the experimental cell velocity and

volume data over the whole time-course of electrohemolysis including both the prehemolytic and hemolytic phases (Figs. S8 and S9). At the stage of the fast hemolytic motion, however, the theoretical curves in Fig. S9 reveal only a little difference between the gOEM (red solid curves) and the minimal elastic model (blue dashed curves). This result indicates that the osmotic water influx during hemolysis affects only slightly the hemolytic cell motion, which in turn justifies the use of the minimal elastic model for modeling the propulsive behavior of hemolyzing RBCs (Fig. 5C).

## Time scale of the diffusive leakage of Hb through a hemolytic hole

As argued in the Discussion section, elastic contraction of osmotically stretched NM2 filaments enables rapid bulk expulsion of the Hb-rich cytosol through a hemolytic hole. We found that ~55% of Hb are expelled from a hemolyzing RBC by the NM2-contraction mechanism within $\Delta t_{\mathrm{hem}} \approx 1$ s after the onset of hemolysis. For comparison, we assess in this section the time required for the passive diffusive leakage of 55% Hb ($\Delta t_{\mathrm{diff}}$) through a single hemolytic hole.

The estimation of $\Delta t_{\mathrm{diff}}$ is based on the following assumptions: (1) The intracellular Hb concentration $C_{\mathrm{Hb}}$ is uniform. $C_{\mathrm{Hb}}$ changes upon Hb diffusion to the extracellular space. (2) The extracellular Hb concentration is zero. (3) The quasi-static diffusion in the hemolytic hole is assumed and the diffusion coefficient of Hb is constant. (4) The pore area $S_{pore} = \pi r_{pore}^2$, where $r_{\mathrm{pore}}$ = 3.1-10 nm is the pore radius. Membrane thickness $\delta$ is ~5 nm and the RBC volume $V_{\mathrm{RBC}}$ =160 fL (swollen spherocyte).

The ruling equation for $C_{\mathrm{Hb}}$ can be expressed as:

$$\frac{dC_{\mathrm{Hb}}}{dt} V_{\mathrm{RBC}} = -D_{\mathrm{Hb}} \frac{dC_{\mathrm{Hb}}}{dx} S_{\mathrm{pore}} \approx -D_{\mathrm{Hb}} \frac{C_{\mathrm{Hb}}}{\delta} S_{\mathrm{pore}} \qquad \text{(S29)},$$

where $D_{\mathrm{Hb}}$ is the diffusion coefficient of Hb (*see* below).

Defining the time interval required for $C_{\mathrm{Hb}}$ to become ~45% of its initial value as $\Delta t_{\mathrm{diff}}$, Eq. S29 can be solved by integration:

$$\int_{C_{\mathrm{Hbini}}}^{0.45C_{\mathrm{Hbini}}} \frac{dC_{\mathrm{Hb}}}{C_{\mathrm{Hb}}} = \int_0^t -\frac{D_{\mathrm{Hb}} S_{\mathrm{pore}}}{\delta \cdot V_{\mathrm{RBC}}} dt\,; \qquad ln(0.45) = -\frac{D_{\mathrm{Hb}} S_{pore}}{\delta \cdot V_{\mathrm{RBC}}} \Delta t_{\mathrm{diff}} \qquad \text{(S30)}$$

$$\Delta t_{\mathrm{diff}} = -ln(0.45) \frac{\delta \cdot V_{\mathrm{RBC}}}{D_{\mathrm{Hb}} S_{\mathrm{pore}}} \qquad \text{(S31)}.$$

Since $D_{\mathrm{Hb}}$ strongly depends on $C_{\mathrm{Hb}}$, we use two different $D_{\mathrm{Hb}}$ values $D_{\mathrm{Hb1}}=3.4 \cdot 10^{-12} m^2/s$ reported for $C_{\mathrm{Hb}}$ = 35 g/dL and $D_{\mathrm{Hb2}}=52 \cdot 10^{-12}$ $m^2s$ reported for a lower $C_{\mathrm{Hb}}$ = 4.9 g/dL (Adams and Fatt, 1967).

Substitution of the two $D_{Hb}$ values into Eq. S31 yields the following range of $\Delta t_{diff} \approx$ 410-6200 s for a hemolytic hole radius $r_{pore}$ = 3.1 nm. Increasing $r_{pore}$ to 10 nm decreases $\Delta t_{diff}$ to 40-600 s. Accordingly, the Hb efflux passive diffusion is slower by 2-3 orders of magnitude than the convective Hb expulsion via the NM2-contraction mechanism.

## Cell-swelling mediated stretching of an NM2 filament attached to the base of a spicule

Assume two states of an RBC, which are an echinocyte with only one spicule (Fig. S10A) and a swollen spherocyte (Fig. S10B). The spicule area in the echinocyte (gray color in Fig. S10A) $A_{spic} = 2\pi a_{spic}^2$ is equal to that of a hemisphere of diameter $L_{NM2} = 2 \cdot a_{spic}$, where $L_{NM2}$=420 nm (red line in Fig. S10A) is the length of a relaxed NM2 filament and $a_{spic}$=210 nm is the spicule radius.

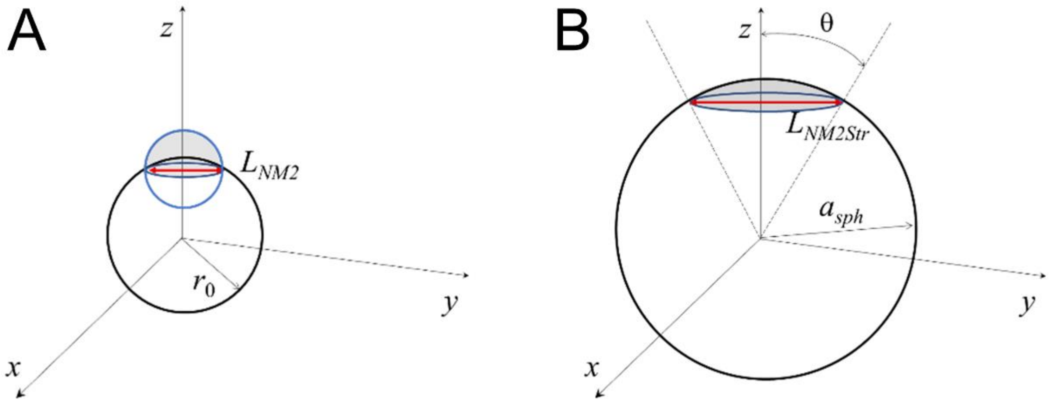


**Figure S10.** Schematics of the swelling-mediated stretching of an NM2 filament (red line) attached to the spicule base. **A**) Simplified echinocyte with only one spicule (gray). A relaxed NM2 filament is connected in a chord-like manner to membrane-bound actins. **B**) During transition from echinocyte to spherocyte, the radius of spicule curvature increases, approaching that of the cell body, which in turn leads to the stretching of the underlying NM2 filament.

During the swelling-mediated transition from echinocyte to spherocyte with a critical volume $V_{crit}$=160 fL (Fig. S10B), the radius of the spicule curvature approaches that of the main cell body, *i.e.*, spherocyte: $a_{sph}$= 3.39 μm. Since the bilayer is nearly inextensible the spicule area $A_{spic}$ remains constant during echinocyte-to-spherocyte transition. Accordingly, $A_{spic}$ is equal to the area of the spherical cap within the polar angle $\theta$, as illustrated by the gray area in the spherocyte in Fig. S10B. Thus, the following relations hold:

$$A_{\text{spic}}^{\text{echinocyte}} = 4\pi(L_{NM2}/2)^2/2 = 2\pi a_{spic}^2 \qquad \text{(S32)}$$

$$A_{\text{spic}}^{\text{spherocyte}} = \int_0^{\theta}\int_0^{2\pi} a_{\text{sph}}^2 \, sin\,\theta\,' d\theta d\theta' = 2\pi a_{\text{sph}}^2[-cos\,\theta\,']_0^{\theta} = 2\pi a_{\text{sph}}^2(1 - cos\,\theta) \qquad \text{(S33)}$$

Since $A_{\text{spic}}$ is constant, $A_{spic}^{echinocyte} = A_{spic}^{spherocyte}$ the following relation for the polar angle $\theta$ can be derived from Eqs. S32 and S33:

$$\theta = arccos\{1 - (a_{spic}/a_{sph})^2\} \qquad \text{(S34)}$$

Since $L_{NM2}$ =2$a_{spic}$ and $a_{sph}$ = 3.39 µm are given, the length of a stretched NM2 filament $L_{NM2str}$, can be expressed as:

$$L_{NM2}^{str} = 2a_{sph}\, sin[\,arccos\{1 - (a_{spic}/a_{sph})^2\}] \qquad \text{(S35)}$$

With an increase of the spicule curvature radius from $a_{spic}$ =0.21 µm to $a_{sph}$ =3.39 µm, the length of a stretched NM2 filament can be calculated as $L_{\text{NM2str}}$ =2$a_{\text{sph}}$×sinθ ≈590nm.

## 3. SUPPLEMENTARY MOVIES

1) **Movie S1:** Self-propelled motion of human RBCs during hemolysis triggered by electroporation.

2) **Movie S2:** Movie corresponding to Fig. 2D1-2D3.

3) **Movie S3**: Movie corresponding to Fig. 3.

4) **Movie S4:** Electroporation in the presence of 250 µM ATP and 70 µM blebbistatin.
